# Isolation of a Novel Jumbo Bacteriophage Effective Against *Klebsiella aerogenes*

**DOI:** 10.3389/fmed.2020.00067

**Published:** 2020-02-28

**Authors:** Rhea Lewis, Adam G. Clooney, Stephen R. Stockdale, Colin Buttimer, Lorraine A. Draper, R. Paul Ross, Colin Hill

**Affiliations:** ^1^APC Microbiome Ireland, University College Cork, Cork, Ireland; ^2^School of Microbiology, University College Cork, Cork, Ireland

**Keywords:** phage therapy, antimicrobial resistance, bacteriophage, biofilm, *Staphylococcus aureus*, phage cocktail, jumbo phage

## Abstract

Increasing levels of bacterial resistance to many common and last resort antibiotics has increased interest in finding new treatments. The low rate of approval of new antibiotics has led to the search for new and alternative antimicrobial compounds. Bacteriophages (phages) are bacterial viruses found in almost every environment. Phage therapy was historically investigated to control bacterial infections and is still in use in Georgia and as a treatment of last resort. Phage therapy is increasingly recognized as an alternative antimicrobial treatment for antibiotic resistant pathogens. A novel lytic *Klebsiella aerogenes* phage N1M2 was isolated from maize silage. *Klebsiella aerogenes*, a member of the ESKAPE bacterial pathogens, is an important target for new antimicrobial therapies. *Klebsiella aerogenes* can form biofilms on medical devices which aids its environmental persistence and for this reason we tested the effect of phage N1M2 against biofilms. Phage N1M2 successfully removed a pre-formed *Klebsiella aerogenes* biofilm. Biofilm assays were also carried out with *Staphylococcus aureus* and Phage K. Phage K successfully removed a preformed *Staphylococcus aureus* biofilm. Phage N1M2 and Phage K in combination were significantly better at removing a mixed community biofilm of *Klebsiella aerogenes* and *Staphylococcus aureus* than either phage alone.

## Introduction

Bacteriophages (phages) are viruses that require bacteria as a host for replication. Studies suggest that in many environments phages outnumber bacterial and archaeal cells 25:1 (1). Phages have been studied using culture based techniques since their independent discovery by Twort and D'Herelle in 1915 and 1917, respectively. More recently, phages have been studied using culture independent methods such as the sequencing of environmental metagenomes which include bacteria, eukaryotic viruses, and phages but also in studies of the virome alone (2). Interest has been growing in the role of phages in general human health (3) but also in specific conditions such as IBD (4). Despite the rise in viral metagenomics, cultivated phages are required for the development of interventions for use in phage therapy (5), food safety (6), and molecular biology (7).

The acronym ESKAPE has been applied to six bacterial pathogens that are of concern due to their ability to cause hospital acquired infections and the difficulty in treating them due to antibiotic resistance (8). The ESKAPE pathogens have shown increased incidence as causative agents in human disease and have increased antimicrobial resistance and negative outcomes. In 2017 the WHO compiled a global priority pathogens list prioritizing the development of new antibiotics (9). The pathogens were chosen based on 10 criteria; mortality, association with issues in healthcare settings and communities, the frequency of resistance, a 10-year trend of resistance, ease of transmission, how they could be prevented in hospital and community settings, and their current treatment. This list included critical priority targets carbapenem-resistant *Acinetobacter baumannii*, carbapenem-resistant *Pseudomonas aeruginosa* and carbapenem-resistant and 3rd generation cephalosporin-resistant Enterobacteriaceae (including *Klebsiella pneumoniae, Escherichia coli, Enterobacter* spp., *Serratia* spp., *Proteus* spp., and *Providencia* spp., *Morganella* spp.). *Klebsiella aerogenes* was previously classified as *Enterobacter aerogenes* (10).

The rise in antibiotic resistance has led to a need for new antimicrobial compounds; however, the number of drug applications and approvals for antibiotics has dropped significantly (11). For example, 19 new antibiotic applications were lodged in the early 1980s but only one was lodged between 2010 and 2012. This shortage of newly developed antibiotics has led to research into alternative antimicrobials (12). Western societies lost interest in phage therapy during World War II with the discovery of antibiotics but interest has been renewed due to the need for new antimicrobials. Phage therapy is currently used in Georgia against a range of pathogens, including *Shigella sonnei, Escherichia coli, Staphylococcus aureus*, and *Pseudomonas aeruginosa*. Randomized controlled trials using phage therapy have been carried out such as PhagoBurn to treat burn wounds infected by *Pseudomonas aeruginosa* and the use of Biophage-PA to treat chronic otitis caused by antibiotic-resistant *P. aeruginosa* (13, 14). In the food industry, consumer distrust has increased toward chemical and artificial preservatives, leading to increased interest in alternative natural methods (15). Phages can also be used in food safety to reduce contamination from livestock during production, disinfect plants, and equipment, reduce bacterial load on food at processing and during storage.

Much work has been carried out isolating phages from different sources. Feces is a common source of phages, such as from ducks (16), cattle (17), pigs (18), and from sewage (19). Phages have been isolated from human sources, such as saliva (20), sputum samples, catheter tips, and pleural effusions (21). Phages have also been isolated from environmental samples, such as seawater, from river estuaries (22), Arctic sea ice and melt ponds (23), soil (24), and yogurt and cheese factories (25).

The aim of this study was to isolate and characterize phages that could potentially be used for phage therapy or in food safety. A total of 163 bacterial strains were isolated from a healthy human fecal sample and used in conjunction with a bank of 79 known bacterial strains as prospective hosts for phages. These strains were screened for phages against a human fecal filtrate and environmental samples collected from a nearby farm. Farm areas tested for phages were from cow slurry (*n* = 2), sheep feces (*n* = 2), maize silage (*n* = 1), and grass silage (*n* = 1). A selection of phages were sequenced and analyzed. One phage isolated on a *Klebsiella aerogenes* host, named phage N1M2, was chosen for further study as it had not been previously sequenced or described. Genomic and phylogenetic analysis revealed it to be a highly novel jumbo phage distantly related to *Pseudomonas* phage OBP, a phage previously described to possess homology to the PhiKZ-like jumbo phages (26).

Medical device-related infections are associated with increased morbidity, mortality, and healthcare costs. Biofilms, which are commonly associated with medical devices, require treatment with antimicrobials in higher levels than planktonic cells (27). Phages have been investigated in human cases and mouse models of medical device infections. For example, phage coating significantly reduced the bacterial load and inflammation in a mouse model of MRSA orthopedic implant infection (28). Moreover, phage significantly reduced bacterial numbers in a mouse model of implant infection caused by biofilm forming *P. aeruginosa* (29). Combinations of phage and antibiotics have also been successful in the treatment of a recurrent infection associated with a left ventricular assist device (30). In a case study, a combination of phage and antibiotics successfully treated a multidrug-resistant *P. aeruginosa* periprosthetic joint infection (31). The rationale behind the inclusion of phage was to aid in the breakdown of a biofilm matrix on the implant.

Urinary tract infections are a common nosocomial infection. *K. aerogenes* strains capable of biofilm production have been isolated from urinary catheters (32), and has also been found to be the causative agent in central venous catheter-related bloodstream infections (33). The effectiveness of phage N1M2 was therefore investigated in biofilms. Mixed community infections are commonly associated with medical devices. Hola et al. found 465/534 urinary catheters were colonized by more than one bacterial strain (32). For this reason mixed community biofilms were also investigated. *Staphylococcus aureus* was chosen because it is one of the most commonly isolated bacteria from catheter-related urinary tract infections and bloodstream infections and methicillin-resistant *S. aureus* (MRSA) is one of the most feared hospital acquired infections (27). The antimicrobial potential of Phage K against *S. aurerus* is well-documented (34). Phage K belongs to the family of *Herelleviridae* and falls within the genus of *Kayvirus* (35). Given the data to date more intensive research into the effectivity of phages in nosocomial infection is warranted especially given the current status of antimicrobial resistance to commonly used antibiotics.

## Methods

### Isolation of Bacterial Hosts

A healthy human fecal sample (1 g) was suspended in 5 ml of 1 × phosphate buffered saline (PBS) and 5 ml 40% glycerol and homogenized by vortexing for 5 min. Bacterial strains were isolated by diluting the fecal suspension in PBS and spread plating 100 μl of dilutions 10^−3^-10^−8^ on MacConkey agar (Merck), Violet Red Bile agar (Merck), Brain Heart Infusion agar (Oxoid), MRS agar (Oxoid), and LB agar (Oxoid). Agar plates were incubated at 37°C aerobically, and also under facultative anaerobic conditions using a gas jar with an Anaerocult A (Merck). Colonies of different morphology, size, and color were isolated from the various different agar plates. Strains were streaked on their respective agar plates in triplicate to purify cultures for phage screening. A bank of known strains was also selected to be used for screening (Supplementary Table 1). Bacterial strains were identified by 16S rRNA sequencing analysis using BLASTn. The 16S rRNA DNA was amplified by colony PCR using primers: F8-Fw: 5′-AGAGTTTGATCMTGGCTC-3′ and R1509-Rv: 5′-GNTACCTTGTTACGACTT-3′.

### Isolation and Purification of Phages

Human fecal and farm environmental samples (2 cow slurry, 2 sheep feces, 1 maize silage, and 1 grass silage) were collected and lysates were prepared immediately. Supernatants were prepared from samples using the following method. Sample (1 g) was suspended in 10 ml of SM buffer (50 mM Tris-HCl; 100 mM NaCl; 8.5 mM MgSO_4_; pH 7.5). Samples were homogenized by vortexing for 5 min, before centrifuging twice at 4,700 × g for 10 min at 4°C in a swing-bucket centrifuge to remove large particulates and bacterial cells. Supernatants were filtered twice through a 0.45 μm pore diameter filter. Bacterial strains were grown overnight in BHI at 37°C with shaking (for aerobes) or without shaking (for facultative anaerobes). The overlay method was used for spot and plaque assays. BHI (1% agar w/v) was used as the base agar in a 100 mm × 15 mm petri dish. Plaque assays were performed by adding 400 μl of 1 M CaCl_2_ (final concentration 10 mM), 100 μl of phage lysate and 100 μl of an overnight culture of bacterial host to 4 ml of soft BHI agar (0.5% agar w/v) kept at 50°C. This mixture was poured on top of the BHI (1% agar w/v) base agar and allowed to solidify. Spot assays were performed by adding 400 μl of 1 M CaCl_2_ (final concentration 10 mM) and 200 μl of an overnight culture of bacterial host to 4 ml of soft BHI agar (0.5% agar w/v) kept at 50°C. This mixture was poured on top of the BHI (1% agar w/v) base agar and allowed to solidify. Ten microliter of phage sample lysates were pipetted on the agar and allowed to dry. Plates were incubated at 37°C for 24–48 h. Single plaque purification by propagation of a single plaque and plaque assay was carried out three times.

### Viral DNA Extraction, Amplification, Library Preparation, and Sequencing

A 20 ml phage lysate with 4 ml of 2.5 M NaCl and 50% polyethylene glycol (PEG) solution (final conc 0.4 M NaCl and 8% (w/v) PEG) added was stored at 4°C on ice overnight. Samples were centrifuged at 4,700 × g for 20 min at 4°C in a swing bucket rotor. Supernatants were removed and pellets were dried for 5 min by inverting tube. Pellets were resuspended in 400 μl SM buffer (50 mM Tris-HCl; 100 mM NaCl; 8.5 mM MgSO_4_; pH 7.5). Forty microliter of 10 × Nuclease Buffer (50 mM CaCl_2_; 10 mM MgCl_2_), was added and treated with 20 U of DNase I and 10 U of RNase I (final concentrations; Ambion) for 1 h at 37°C. Nucleases were inactivated at 70°C for 10 min before samples were treated with 2 μl of freshly prepared 20 mg/μl Proteinase K for 20 min at 56°C. Phage DNA extractions were performed using Norgen BioTek Corp Phage DNA Isolation Kit as described by the manufacturer starting at addition of Lysis Buffer B. The Elution Buffer (50 μl) was passed through the column twice to maximize DNA recovery. Viral DNA concentrations were equalized before paired-end Nextera XT library preparation (Illumina, San Diego, CA, USA) as described by the manufacturer. Metagenomic sequencing was performed using the Illumina MiSeq (Illumina Inc., San Diego, CA, USA) by generating 300 bp paired-end read libraries following the manufacturer's instructions.

### Transmission Electron Microscopy

Phage lysate was concentrated by ultracentrifugation at 40,000 RPM for 2 h at 4°C. One hundred and eighty milliliter of phage lysate was concentrated to 6 ml. The resulting phage preparation was placed on to a CsCl step gradient composed of 1.3, 1.5, and 1.7 g/ml layers and ultracentrifuged at 40,000 RPM for 3 h at 4°C. Resulting phage bands were collected and subjected to dialysis with two changes of SM buffer at 4°C. Five microliter aliquots of the viral fraction were applied to Formvar/Carbon 200 Mesh, Cu grids (Electron Microscopy Sciences) with subsequent removal of excess sample by blotting. Grids were then negatively contrasted with 0.5% (w/v) uranyl acetate and examined at UCD Conway Imaging Core Facility (University College Dublin, Dublin, Ireland) by transmission electron microscope.

### Bacterial DNA Extraction, Library Preparation, and Sequencing

N1 DNA was extracted from an overnight culture using a Qiagen DNeasy Blood and Tissue kit as per the manufacturer's instructions with pre-treatment for Gram-negative bacteria. N1 DNA was sequenced using the Oxford Nanopore MinION as per manufacturer's instructions.

### Phage Propagation and Plaque Assays

After isolation of phage N1M2 propagation was carried out in Tryptic Soy Broth (TSB) with calcium boroglucinate (final concentration 10 mM). Plaque assays were carried using 1% Tryptic Soy Agar (TSA) base agar and 0.4% TSA overlay with calcium boroglucinate (final concentration 10 mM). Phage N1M2 host range was established on 1.5% LB base agar and a 0.2% LB agarose overlay with CaCl_2_ (final concentration 10 mM) by carrying out plaque assays against a range of bacteria. The efficiency of plaquing was calculated by dividing the titer of phage N1M2 on the strain to be tested by the titer of phage N1M2 on strain N1. For host range assay bacteria were grown overnight in LB broth at 37°C under aerobic conditions. Phage K propagation was carried out in TSB with calcium boroglucinate (final concentration 10 mM). Phage K plaque assays were carried using 1% TSA base agar and 0.4% TSA overlay with calcium boroglucinate (final concentration 10 mM).

### N1 Bioinformatic Analysis

*Klebsiella aerogenes* N1 draft genome and *K. aerogenes* KCTC 2190 genome were compared using Easyfig v2.2.2 (36). Genomes were compared using BLASTn with default settings except for minimum length and minimum identity which were changed to 100 bp and 50%, respectively. PHASTER was used to predict possible prophage regions in N1 and KCTC 2190 (37). JGI IMG/M genome browser was used to analyze function of KTCT 2190 genes (38).

### Phage N1M2 Bioinformatic Analysis

Sequencing reads of phages were quality filtered and assembled into contigs using SPAdes meta (39). BLASTn NT database was used to ensure the phage N1M2 sequence was not similar to that of a known phage (40). Phage N1M2 genome was annotated using VIGA [https://github.com/EGTortuero/viga (41)]. Functional annotation of ORF gene products and amino acid identity was established using BLASTn and InterProScan (42). Transmembrane helices in proteins were predicted using TMHMM (43). LipoP (44) was used for predictions of lipoproteins. The molecular weights and isoelectric points of the predicted ORFs were determined using https://web.expasy.org/compute_pi/. Amino acid and codon usage statistics were determined using the University of Georgia online tool (http://www.cmbl.uga.edu/software/codon_usage.html). The presence of transfer RNA genes was determined using tRNAscan-SE [http://lowelab.ucsc.edu/tRNAscan-SE/ (45)] and ARAGORN (http://130.235.46.10/ARAGORN/ (46)]. BLASTn search (evalue cut-off 1e-10) was performed against NCBI nt database and bacterial section of NCBI RefSeq genomes database release 89 to align to bacterial tRNAs. Potential Rho-independent terminators were identified using ARNold [http://rna.igmors.u-psud.fr/toolbox/arnold (47)] and then confirmed with MfoldQuikFold [http://unafold.rna.albany.edu/?q1/4DINAMelt/Quickfold (48)] using RNA energy rules 3.0. Potential promoters were identified by extracting sequences 100 bp upstream of each ORF using FeatureExtract 1.2L (light) Server [http://www.cbs.dtu.dk/services/FeatureExtract/ (49)] and submitting these sequences to MEME (Multiple Em for Motif Elicitation) [http://meme-suite.org/tools/meme (50)]. The annotated genome was visualized using GView (51). Multiple sequence alignment was carried out with MEGA7 (52) using MUSCLE (53). Phylograms were then constructed using the major capsid protein, the large terminase and DNA polymerase being used among phage N1M2 and other jumbo phages. The evolutionary history of phage N1M2 was inferred by using the Maximum Likelihood method based on the JTT matrix-based model (54) in MEGA 7. The bootstrap consensus tree inferred from 1,000 replicates was taken to represent the evolutionary history of the taxa analyzed (55). The whole genome phylogenetic trees based on nucleotides and amino acid sequences were generated by Victor (56) using the Genome-BLAST Distance Phylogeny (GBDP) (57) method under settings recommended for prokaryotic viruses. Bacterial CRISPR spacer database (made in house using PILER-CR v1.06 from NCBI RefSeq genomes database, release 89) was used as a query for BLASTn search (evalue cut-off 1e-5) against the phage genome.

### Biofilm Assays

Ninety-six-well plates were filled as follows based on Dalmasso et al. (20). Each well was filled with 200 μl TSB with or without 1% glucose inoculated at 1% with an overnight culture of the relevant strain. For mixed biofilms 1% of an overnight culture of N1 and 1% of an overnight culture of DPC 5247 (Methicillin sensitive) was added. Plates were incubated at 37°C for 48 h to allow the biofilm to form. Broth containing planktonic cells was carefully removed to avoid disruption of the formed biofilm. Hundred microliter TSB containing 20 mM calcium boroglucinate and 100 μl of phage dilution was added to each well. SM buffer was added to control wells. Plates were incubated at 37°C for 48 h as indicated. Twenty-four hour biofilm formation was also tested as was phage addition for 72 h. For investigating stopping the formation of a biofilm 50 μl phage was added at the same time as the 200 μl overnight culture of the relevant strain. After incubation, broth containing planktonic cells was carefully removed. Wells were washed twice with 150 μl phosphate buffered saline (PBS). An XTT/menadione assay was carried out as follows. XTT/menadione solution was prepared by adding 0.01 g XTT to filter sterilized water and filter sterilizing using a 0.22 μm filter. 0.027 g menadione was added to 10 ml acetone. Ten microliter of menadione acetone solution was added to 20 ml of XTT solution. Hundred microliter of XTT/menadione solution was added to each well of the 96-well plate and incubated at 37°C in the dark for 2 h. The absorbance was then measured at a wavelength of 492 nm in a plate reader. The XTT/menadione assay relies on the reduction of tetrazolium salt 2,3-bis[2-methyloxy-4-nitro-5-sulfophenyl]-2H-tetrazolium-5-carboxanilide (XTT) by metabolically active cells to an orange/yellow water-soluble formazan derivative that can be quantified colorimetrically meaning only live cells are counted (58).

N1 and DPC 5247 numbers were quantified in a mixed biofilm. The experiment was carried out once but counts were carried out in duplicate. Biofilms were formed and treated as previously described. After incubation, broth containing planktonic cells was carefully removed. Wells were washed twice with 150 μl PBS. Two hundred microliter PBS was added to each well and mixed by pipetting up and down. All the wells in a row were combined and serially diluted 1:10 in PBS. Hundred microliter of the relevant dilution was spread plated on UTI ChromoSelect agar. Plates were incubated at 37°C for 24 h.

### Statistical Analysis

For XTT assays 2 rows per 96-well plate were used per condition to be tested and all experiments were independently performed 3 times. For enumeration of N1 and DPC 5247 the experiment was carried out once but counts were carried out in duplicate. Graphs were prepared using GraphPad Prism and are presented as mean values of a single experiment that represent the trend seen in the triplicate experiment. Error bars in the figures indicate standard error of the mean. One way ANOVA was used to determine the significance of differences between controls and treated samples.

## Results

### Genome Information of Strain N1

**Figure 1:**
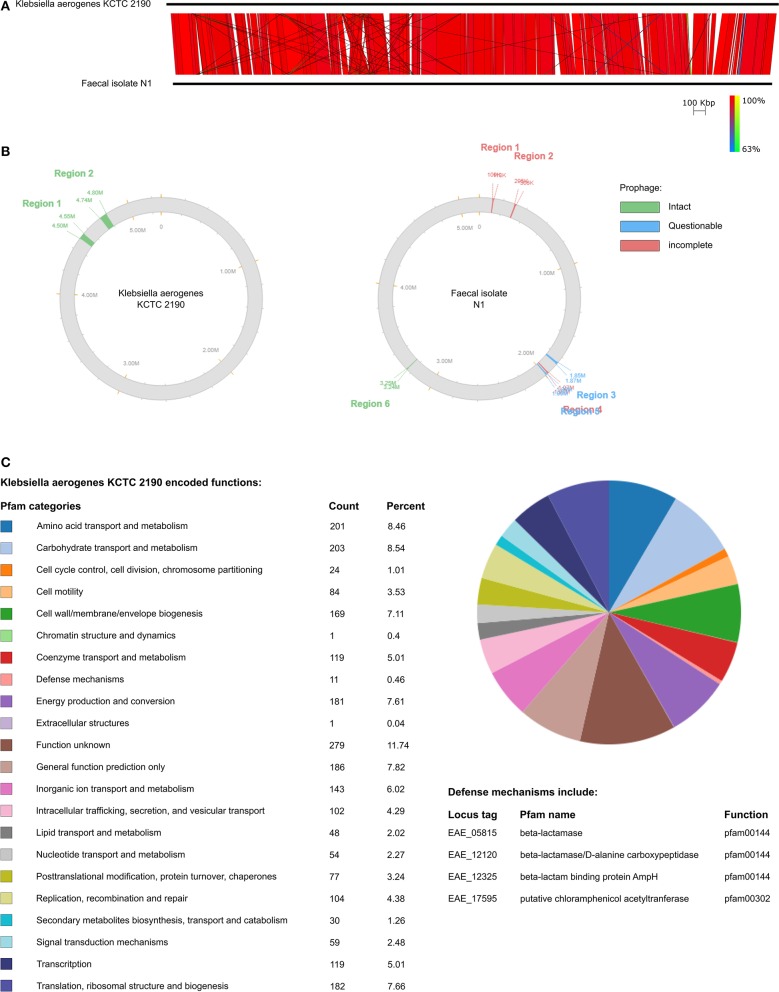
**(A)** Comparison of N1 draft genome and *K. aerogenes* KCTC 2190. **(B)** Prophages predicted using PHASTER in N1 and *K. aerogenes* KTCT 2190. **(C)** Pfam predicted functions of *K. aerogenes* KTCT 2190 genes.

Bacterial strain N1 was isolated from a healthy human fecal sample using MacConkey agar and identified as *Klebsiella aerogenes* by 16S rRNA sequencing. It was then genome sequenced using the Oxford Nanopore MinION. This was classed as a draft genome due to the high error rate associated with MinION sequencing (59). Strain N1 has a chromosome of 5,167,877 bp with a GC content of 55.3%. The chromosome encodes 14,509 predicted open reading frames (ORFs) ranging in size from 90 to 3,315 bp. N1 contained two plasmids of 72,172 bp and 51,484 bp with GC contents of 43.6 and 47.2%, respectively. Plasmid 1 encoded 158 ORFs ranging in size from 93 to 636 bp. Plasmid 2 encoded 123 ORFs ranging in size from 114 to 660 bp. No resistance genes or tRNA were predicted in plasmids 1 and 2. Twenty-four tRNA genes were predicted in the chromosome (Table 1). N1 was compared to the reference genome *Klebsiella aerogenes* KCTC 2190. KCTC 2190 and N1 are similar in gene order with no inversions or large areas of difference (Figure 1A). Two intact prophage regions were predicted in KCTC 2190 while one intact, two questionable, and two incomplete prophage regions were predicted in N1 (Figure 1B). The Pfam categories of KCTC 2190 encoded functions were used as indicators of the functions associated with N1 due to their similarity (Figure 1C). Almost 12% of KCTC 2190 genes were of unknown function which is a common occurrence. KCTC 2190 included a number of antibiotic resistance genes and a number of different antibiotic resistance genes were predicted in the N1 genome confirming its importance in the rise of antibiotic resistance and as an ESKAPE pathogen (Supplementary Table 2). Genes encoding MarR, EmrR, and AcrA are present in N1 and all have been linked to multidrug resistance in Enterobacteriacae (60). AcrA has been associated with resistance to carbapenem antibiotics. A number of efflux pumps were present in N1 and these are a common mechanism of antibiotic resistance in *Klebsiella aerogenes*.

**Table 1:** Codon usage and tRNA present in phage N1M2 and N1.

	**Codon**	**N1M2 frequency %**	**N1 frequency %**	**Ratio (phage/host)**	**N1M2 tRNA (location)**	**N1 tRNA (location)**
Phage < Host	GCC	0.84	3.29	0.26	0	3 (569533–569608) (569682–569758) (569832–569907)
	GCG	0.57	4.13	0.14	0	0
	CGA	0.29	0.82	0.35	0	0
	CGC	0.60	3.11	0.19	0	0
	CGG	0.16	1.62	0.10	0	0
	AGG	0.17	0.38	0.45	0	0
	TGC	0.24	1.13	0.21	0	0
	CAG	1.59	2.77	0.57	0	4 (295367–295451) (295480–295563) (295593–295677) (925991–926076)
	GGC	0.84	3.84	0.22	0	0
	GGG	0.77	1.32	0.58	0	0
	CAC	0.74	1.11	0.67	0	0
	CAT	1.04	1.23	0.85	1 (249035–249111)	0
	CTC	0.73	1.23	0.59	0	0
	CTG	2.25	4.95	0.45	0	0
	CCC	0.16	0.83	0.19	0	0
	CCG	0.55	3.10	0.18	0	1 (926231–926307)
	TCC	0.81	1.04	0.78	0	0
	TCG	0.31	1.38	0.22	0	0
	AGC	0.62	2.08	0.30	0	0
	ACC	1.75	2.51	0.70	0	0
	ACG	0.44	1.44	0.31	0	0
	TGG	1.22	1.54	0.79	0	0
	GTC	0.94	1.74	0.54	0	1 (962722–962798)
	GTG	0.84	2.31	0.36	0	1 (926098–926174)
Phage = Host	GAC	1.90	1.83	1.04	0	0
	GAG	1.64	1.75	0.94	0	0
	GGA	0.86	0.86	1.00	0	0
	CTA	0.57	0.60	0.95	0	0
	TTT	1.88	2.08	0.90	0	0
Phage > Host	GCA	1.77	1.25	1.42	0	0
	GCT	2.92	1.63	1.79	0	0
	CGT	2.56	1.66	1.54	0	0
	AGA	0.72	0.39	1.85	0	0
	AAC	3.15	2.08	1.51	0	0
	AAT	2.68	1.41	1.90	0	0
	GAT	4.63	2.79	1.66	0	2 (786427–786503) (966444–966520)
	TGT	0.62	0.53	1.17	0	1 (778986–77906)
	CAA	1.79	1.15	1.56	0	2 (331627–331705) (440212–440130)
	GAA	4.86	2.86	1.70	0	1 (610794–610869)
	GGT	4.15	1.68	2.47	0	1 (778693–778620)
	ATA	0.71	0.58	1.22	0	0
	ATC	3.32	2.60	1.28	0	0
	ATT	3.08	2.15	1.43	0	0
	CTT	1.55	1.01	1.53	0	0
	TTA	1.62	1.02	1.59	0	0
	TTG	1.45	1.27	1.14	0	1 (925945–925873)
	AAA	4.40	2.82	1.56	0	0
	AAG	2.44	1.30	1.88	0	0
	ATG	2.49	2.16	1.15	0	0
	TTC	2.53	1.84	1.38	0	4 (409621–409696) (740942–741019) (778699–778773) (875919–875994)
	CCA	1.58	0.73	2.16	0	1 (962668–962592)
	CCT	1.83	0.70	2.61	0	0
	TCA	0.88	0.78	1.13	0	0
	TCT	2.13	0.73	2.92	1 (249512–249588)	0
	AGT	1.23	0.58	2.12	0	0
	ACA	1.25	0.48	2.6	0	0
	ACT	3.02	0.68	4.44	0	0
	TAC	1.43	1.23	1.16	0	0
	TAT	2.69	1.50	1.79	0	0
	GTA	1.77	0.94	1.88	0	1 (778891–778975)
	GTT	3.40	1.43	2.38	1 (249909–249982)	0

*Codon usage was determined using the University of Georgia online amino acid and codon usage statictics online tool. The presence of transfer RNA genes was determined using tRNAscan-SE and ARAGORN*.

### General Information and Characterization of Phage N1M2

**Figure 2:**
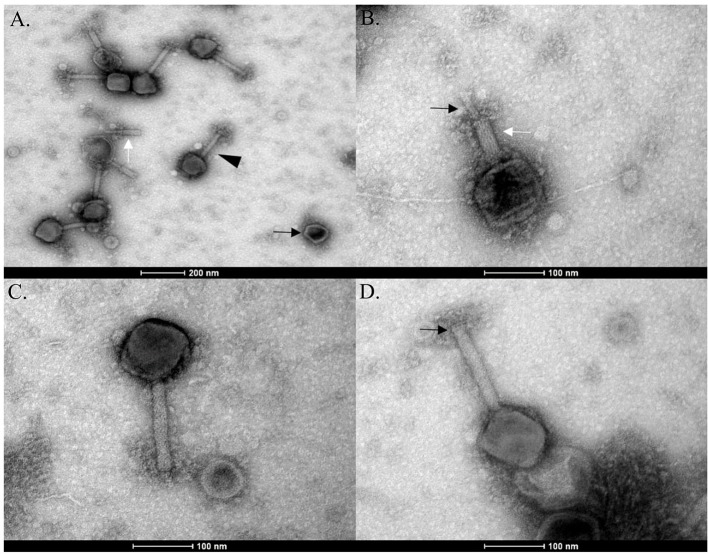
EMs of phage N1M2. **(A)** Empty phage capsid (black arrow), broken phage tail (white arrow), and fully formed phage particle (black triangle). **(B)** Contracted tail sheath (white arrow) with tail core (black arrow). **(C)** Phage N1M2 with uncontracted tail. **(D)** Baseplate (black arrow) and neck structure (white arrow) of uncontracted tail.

Phage N1M2 was isolated against *Klebsiella aerogenes* N1 from maize silage. Phage N1M2 had a genome size of 253,367 bp, placing it among the double stranded DNA jumbo phages. The mean GC content is 40.9%. EM showed phage N1M2 to be a *Myoviridae* phage as evidenced by the long contractile tail (Figures 2A,C). Broken tail and head structures were visible. The contraction of the tail sheath made the tail core visible (Figure 2B). No tail fibers were visible in the EM images (Figure 2D). The average capsid size was 113 nm (±6 nm) × 101 nm (±7 nm) (*N* = 7). The average tail size was 158 nm (±11 nm) × 21 nm (±1 nm) (*N* = 7).

The host range of phage N1M2 was tested against a range of bacterial strains (Table 3). In addition to *Klebsiella aerogenes* phage N1M2 also infected *Klebsiella aerogenes* NCIMB 10102 and *Klebsiella pneumoniae* NCIMB 13218. Plaque morphology was similar on all three strains but the efficiency of plaquing was much lower for Klebsiella pneumoniae NCIMB 13218 compared to N1 and *Klebsiella aerogenes* NCIMB 10102 (0.017 ± 0.009 compared to 1 ± 0 and 0.667 ± 0.17, respectively).

### The Genome of Phage N1M2

The phage N1M2 genome is comprised of 257 ORFs of which 39 are encoded on the minus strand (Supplementary Table 3). The putative gene products ranged in size from 29 to 3,375 amino acids. All ORFs were predicted to start with AUG. Based on a combination of BLAST and InterProScan 85 putative gene products were identified. Of the 172 unidentified putative gene products 106 aligned to unnamed protein products in *Pseudomonas* phage OBP. Of the unnamed protein products of *Pseudomonas* phage OBP the identity ranged from 21 to 88%. Three tRNA genes were predicted and all were contained within a 947 bp region located between 249,035 and 249,982 bp (Table 1). The tRNA genes were similar to those found in *Escherichia* species, *Salmonella enterica, Shigella* species and Stx2-converting phage, and an *Enterobacteria* phage. No integrase, excisionase, or repressor genes were identified, suggesting that the phage follows a lytic lifestyle (Supplementary Table 3).

The potential promoter TBYAWWWWWTTTCARRYAKATATTATYWAAGTGWA was identified at 31 locations in the genome (Supplementary Table 4). The promoter was compared to similar promoters in jumbo phages related to phage N1M2 (Table 2). Fifty-six potential rho-independent terminators were predicted throughout the genome of phage N1M2 (Supplementary Table 5).

**Table 2:** Predicted promoter of phage N1M2 compared to predicted promoters of Pseudomonas phage OBP, Pseudomonas phage 201phi2-1, Pseudomonas phage EL, and Pseudomonas virus phiKZ.

**Phage**	**Promoter sequence**
N1M2	TBYAWWWWWTTTCARRYAKATATTATYWAAGTGWA
OBP	BSHAWWWWWTTTYARRYAKATATTATYWWADTG
2012-1	TTAWTAVAA_HYWTTTRARR_BTATATTACDWHDGTG
EL	WTTTYAAACCTACATTATY
phiKZ	TATATTAC

*Bases colored red match the sequence of the predicted promoter of OBP promoter. Bases colored blue match the sequence of the predicted promoter of phiKZ*.

**Figure 3:**
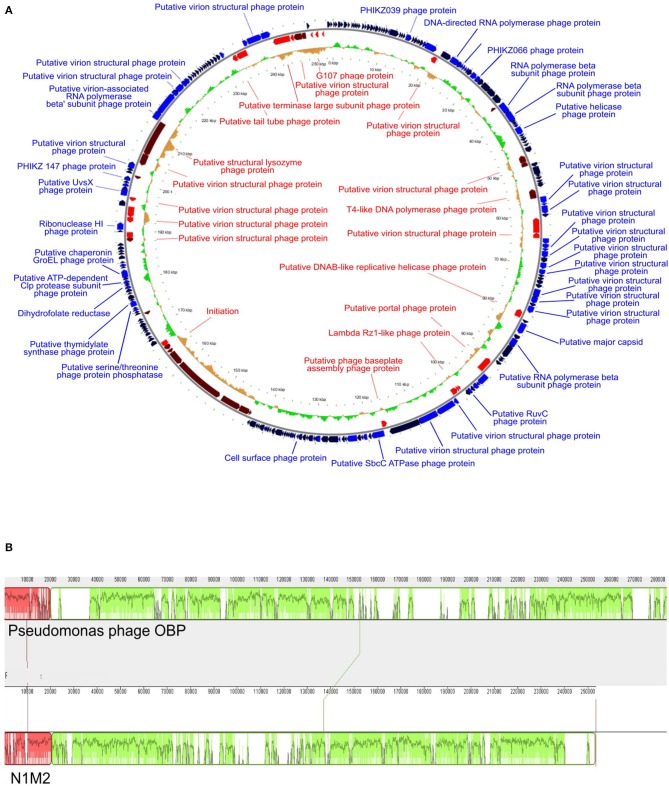
**(A)** Genome map of phage N1M2. On the outer ring blue arrows represent genes on the forward strand, red arrows represent genes on the reverse strand, and black arrows represent hypothetical proteins. The inner ring represents GC skew compared to the average GC content of phage N1M2. **(B)** Mauve alignment of phage N1M2 and Pseudomonas phage OBP.

**Figure 4:**
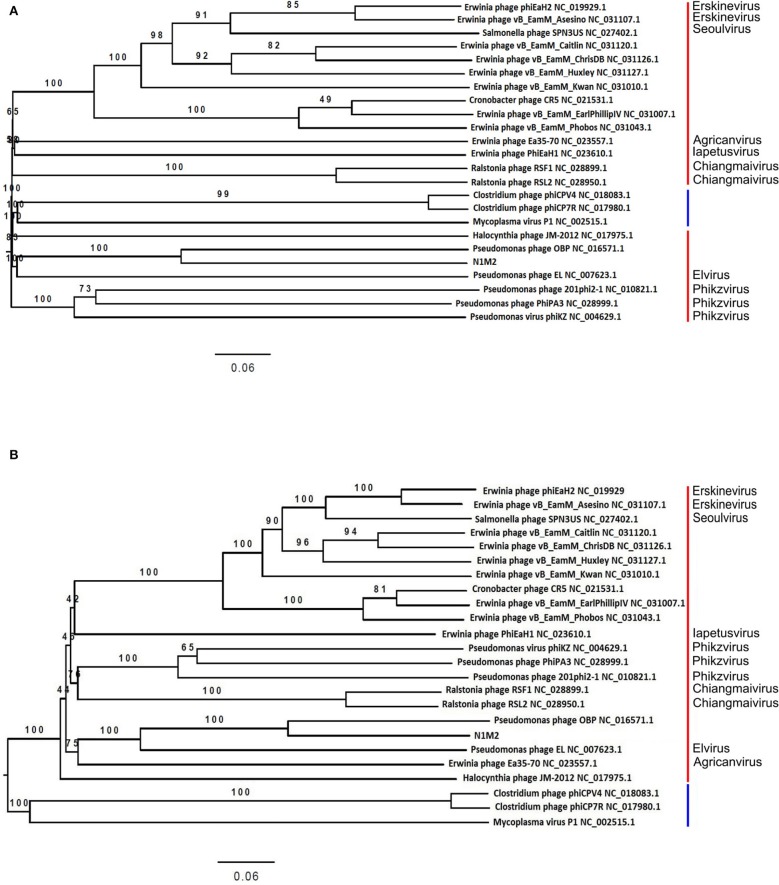
Victor-generated phylogenomic Genome-BLAST Distance Phylogeny (GBDP) trees of *K. aerogenes* phage N1M2 and other jumbo phages inferred. **(A)** Using nucleotide identity and the formula D6 and yielding average support of 88%. **(B)** Using amino acid identity and the formula D6 and yielding average support of 86%. The numbers above branches are GBDP pseudobootstrap support values from 100 replicates. Red bars represent family *Myoviridae*, blue bars represent family *Podoviridae*.

**Figure 5:**
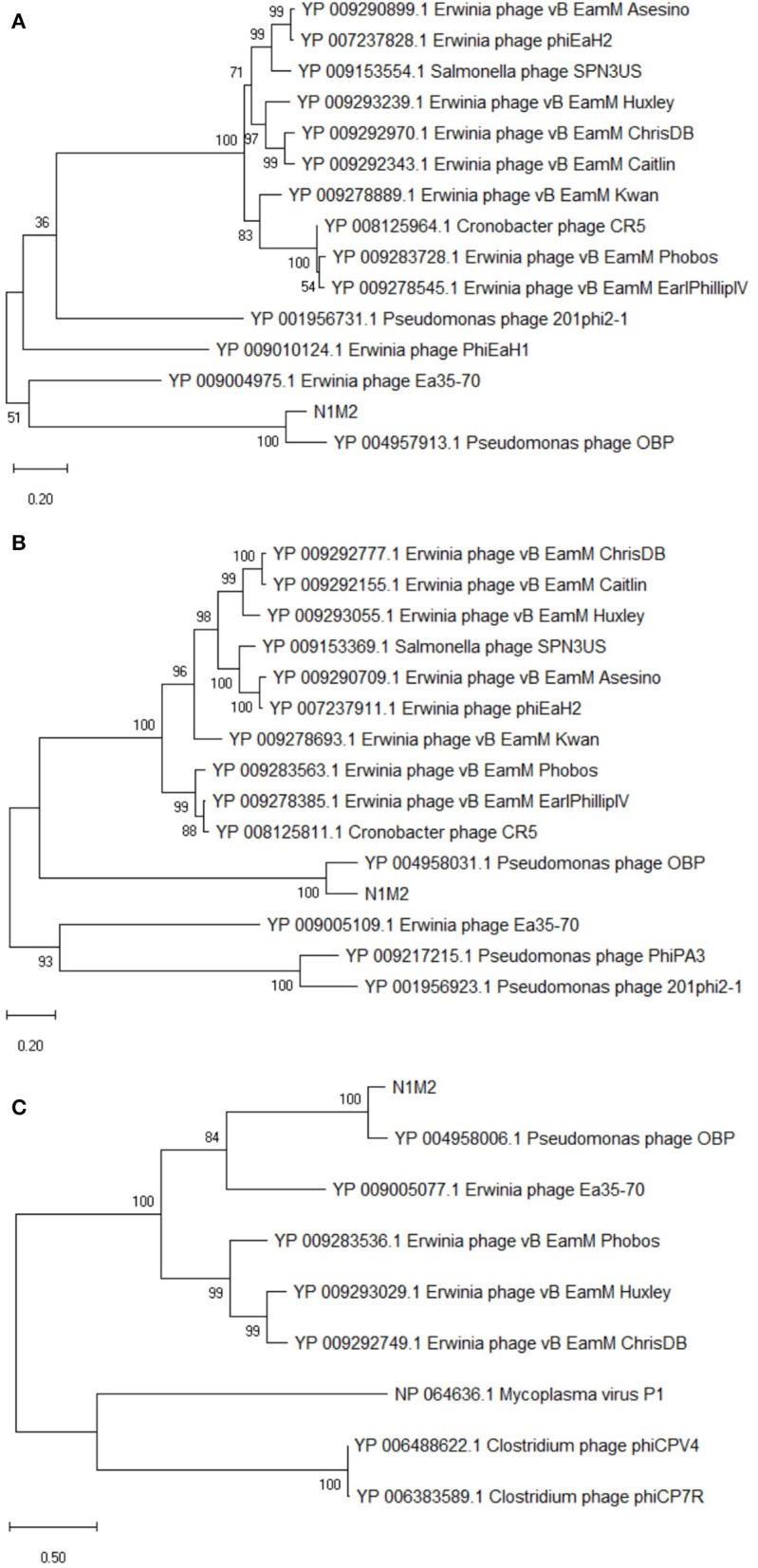
Phylogenetic analysis of amino acid sequences of *K. aerogenes* phage N1M2 and other jumbo phages using maximum likelihood (Whelan and Goldman substitution model), with 1,000 bootstrap replicates of **(A)** major capsid protein, **(B)** large terminase, and **(C)**. DNA polymerase.

At the DNA level phage N1M2 was found to have little homology to phage genomes available on public databases, with the closest match being the aforementioned *Pseudomonas* phage OBP (BLASTn analysis coverage: 55%, identity: 72.73%). The majority of proteins which aligned to known proteins on BLAST aligned to this phage (Supplementary Table 3) (Figure 3B). To investigate the relationship of phage N1M2 to other phages phylogenetic trees were constructed using the nucleotide and amino acid whole genome sequences (Figure 4). To further characterize the relationship between phage N1M2 and some of these phages phylogenetic trees were constructed using the amino acid sequences of the major capsid protein, large terminase and DNA polymerase (Figure 5).

### Single Strain Biofilms Using Strain N1

**Figure 6:**
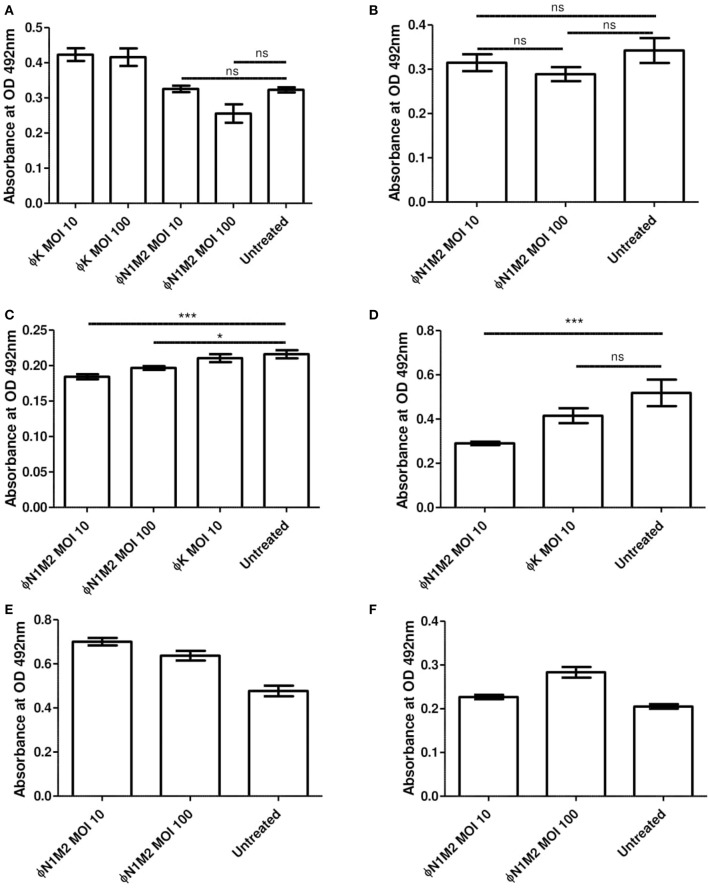
Effect of phage N1M2 on biofilms formed by N1 *K. aerogenes*. Biofilm metabolic activity was assessed by OD492 nm measures after treatment with XTT supplemented with menadione. **(A)** 1% glucose 48 h biofilm, 48 h phage. **(B)** No glucose 48 h biofilm, 48 h phage. **(C)** No glucose 48 h biofilm, 72 h phage. **(D)** No glucose 24 h biofilm, 48 h phage. **(E)** 1% glucose stop biofilm formation over 48 h. **(F)** No glucose stop biofilm formation over 48 h. *P < 0.001; ***P < 0.05.

Biofilms of *K. aerogenes* N1 were formed under various conditions and XTT assays were used to assess the efficiency of phage N1M2 in destroying existing biofilms and preventing biofilm formation (Figure 6). Biofilm experiments were performed three times with duplicate readings and the data from one experiment is presented as a representative of the triplicate results. Phage N1M2 at an MOI of 10 or 100 for 48 h reduced an N1 biofilm formed in the presence of glucose over 48 h but this did not achieve significance (*P* > 0.05) (Figure 6A). Phage K at an MOI of 10 and 100 also had no significant effect on the N1 biofilm (Figure 6A) which was expected since phage K does not infect strain N1. Similarly, phage N1M2 at an MOI of 10 or 100 for 48 h did not significantly reduce an N1 biofilm formed without glucose over 48 h (*P* > 0.05) (Figure 6B). However, phage N1M2 applied at an MOI of 10 or 100 for 72 h significantly reduced an N1 biofilm formed over 48 h without glucose (*P* < 0.001 and *P* < 0.05, respectively) (Figure 6C). The reduction by phage N1M2 at an MOI of 10 or 100 were not significantly different. Phage K at an MOI of 10 applied for 72 h did not reduce the N1 biofilm (Figure 6C). In an N1 biofilm formed over 24 h without glucose phage N1M2 applied at an MOI of 10 for 48 h once again significantly reduced the biofilm (*P* < 0.001) (Figure 6D). Once again Phage K had no effect.

As well as destroying preformed biofilms the ability of phage N1M2 to impede the formation of biofilms was investigated. To do this phage N1M2 was added when the bacteria was inoculated. Phage N1M2 applied at an MOI of 10 did not prevent a biofilm from forming or reduce the level of biofilm formed. This was seen in biofilms formed with (Figure 6E) and without glucose (Figure 6F).

### Single Strain Biofilms Using DPC 5247

**Figure 7:**
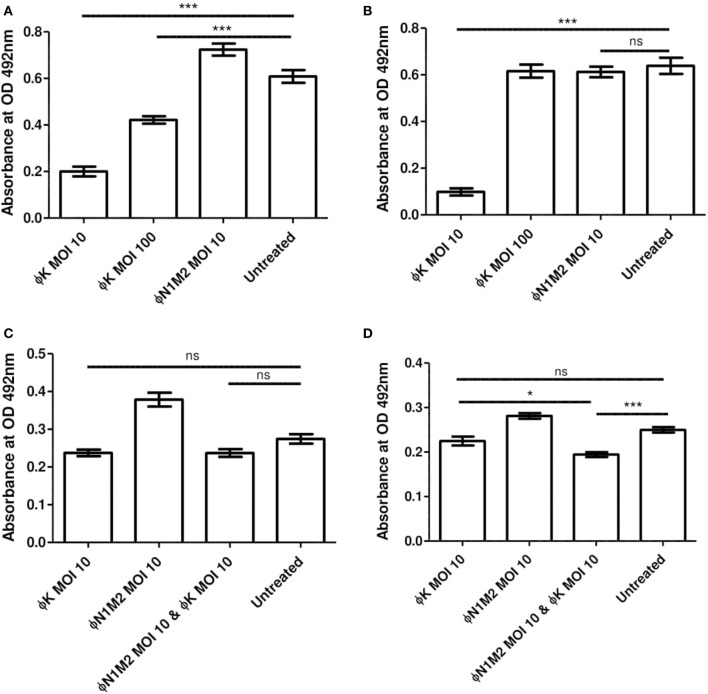
Biofilm metabolic activity was assessed by OD492 nm measures after treatment with XTT supplemented with menadione. **(A)** Effect of Phage K on biofilms formed by DPC 5247 *S. aureus*. 1% glucose 48 h biofilm, 48 h phage **(B)**. Effect of Phage K on biofilms formed by DPC 5247 *S. aureus*. No glucose 48 h biofilm, 48 h phage. **(C)** Effect of phage N1M2 and Phage K on mixed biofilms formed by N1 *K. aerogenes* and DPC 5247 *S. aureus*. 1% glucose 48 h biofilm, 48 h phage. **(D)** Effect of phage N1M2 and Phage K on mixed biofilms formed by N1 *K. aerogenes* and DPC 5247 *S. aureus*. No glucose 48 h biofilm, 48 h phage. *P < 0.001; ***P < 0.05.

Biofilms of *S. aureus* DPC 5247 were formed under various conditions and the impact of Phage K was measured using XTT assays (Figure 7). Phage K at an MOI of 10 or 100 applied for 48 h significantly reduced a DPC 5247 biofilm formed in the presence of glucose over 48 h (*P* < 0.001) (Figure 7A). Phage N1M2 at an MOI of 10 had no effect on the DPC 5247 biofilm (Figure 7A) which was expected since phage N1M2 cannot infect DPC 5247. The reduction due to Phage K at an MOI of 10 was significantly better than that of Phage K at an MOI of 100 (*P* < 0.001). Phage K at an MOI of 10 applied for 48 h also significantly reduced a DPC 5247 biofilm formed without glucose over 48 h (*P* < 0.001) (Figure 7B). Phage N1M2 at an MOI of 10 applied for 48 h had no significant effect on a DPC 5247 biofilm formed without glucose over 48 h.

### Mixed Community Biofilms Using N1 and DPC 5247

Mixed community biofilms of N1 and DPC 5247 were formed and treated with phage N1M2 and Phage K alone and in combination. XTT assays were carried out to assess their effect (Figure 7). Phage N1M2 and Phage K at MOIs of 10, alone and in combination, applied for 48 h did not significantly reduce a mixed biofilm of N1 and DPC 5247 formed in the presence of glucose over 48 h (*P* < 0.001) (Figure 7C). Equally, in a mixed community biofilm of N1 and DPC 5247 grown for 48 h without glucose phage N1M2 alone and Phage K alone at MOIs of 10 applied for 48 h did not significantly reduce the biofilm. However, phage N1M2 and Phage K at MOIs of 10 in combination significantly reduced the biofilm (*P* < 0.001) (Figure 7D).

Untreated and phage-treated biofilms were quantified by dilution and spread plating on UTI ChromoSelect agar (Supplementary Figure 1). N1M2 and Phage K at MOIs of 10, alone and in combination, did not reduce the N1 portion of the community. However, Phage K at an MOI of 10 and N1M2 and Phage K at MOIs of 10 in combination reduced the DPC 5247 members of the community.

## Discussion

Phage N1M2 is the first jumbo phage identified which kills *Klebsiella aerogenes*. Jumbo phages have a number of common properties which differentiate them from smaller phages (61). Jumbo phages are tailed phages with genomes larger than 200,000 bp with large virions. The genes are distributed throughout the genome rather than in organized blocks as is typical of smaller phage genomes. Also jumbo phages contain more genes associated with biological processes such as genome replication, nucleotide metabolism, and lysis of host cell peptidoglycan than smaller phages. Some jumbo phages have more than one DNA polymerase or RNA polymerase gene. Jumbo phages are phylogenetically unrelated to smaller phages. This was evident in our analysis as phage N1M2 showed no similarity to small *K. aerogenes* phages and was most closely related, albeit only distantly, to other jumbo phages (Figure 3). Jumbo phages are isolated more rarely than smaller phages for a number of reasons related to their large size. Jumbo phages often cannot diffuse in semi-solid media meaning they are not always visible as plaques. Their large size also means they can be removed when filtering out bacteria as they cannot pass through smaller filter pores.

The genome of phage N1M2 was suggested to be circularly permuted based on its similarity to *Pseudomonas* phage OBP, its closest relative (26). *Pseudomonas* phage EL, the closest relative of *Pseudomonas* phage OBP, is also circular permuted. When aligned using Mauve, phage N1M2 and OBP showed two areas of similarity, a short region and a long region, although within these similar regions are a number of regions with no homology (Figure 3B). The areas of phage N1M2 that align to OBP ranged from 21 to 88% identity. In the whole genome trees at nucleotide and protein level phage N1M2 and *Pseudomonas* phage OBP formed a separate, strongly bootstrapped clade (Figure 4). The major capsid protein, large terminase and DNA polymerase trees supported these whole genome trees (Figure 5).

A potential promoter TBYAWWWWWTTTCARRYAKATATTATYWAAGTGWA was identified at thirty-one locations in the phage N1M2 genome (Supplementary Table 4). Similar sequences have been identified in OBP and *Pseudomonas* phage 201phi2-1 (26), *Pseudomonas* phage EL (62) and *Pseudomonas* phage phiKZ (63). The promoter found in phage N1M2 was most similar to that of OBP (Table 2). The OBP promoter was suggested as phage specific based on its lack of similarity to sequenced *Pseudomonas* genomes and was deemed to be an early promoter as they were located at the start of operons associated with early genes but not middle or late genes (26). This promoter was situated before ORFs with predicted products with unknown functions but also with predicted products such as RNA polymerase beta subunit, putative virion structural protein, and putative lytic murein transglycosylase.

Despite the clinical importance of *K. aerogenes* little work has been carried out isolating and characterizing *K. aerogenes* phages. There are only four phage genomes publically available: vB_EaeM_φEap-1 (NC_028772), F20 (JN672684) (64), vB_EaeM_φEap-2 (NC_028695) (65), and vB_EaeM_φEap-3 (KT321315) (66). Verthe et al. (67) also isolated UZ1 *K. aerogenes* phage but no genome sequence is available. None of these phages are jumbo phages, ranging from 39,133 to 175,814 bp. The host range of phage N1M2 was tested against a range of bacterial strains selected based on these publications. It is difficult to establish the host range of *K. aerogenes* phages in general based on this small amount of data. So far the host range of *K. aerogenes* phages appears to vary. In some cases it is limited to a single *K. aerogenes* strain, although only a small number of strains were tested (67). In other cases it can include multiple *K. aerogenes* strains but no other genera or species (65, 66). Phage N1M2 infected the two *K. aerogenes* strains tested and also *Klebsiella pneumoniae*, although the efficiency of plaquing of phage N1M2 was much lower on *Klebsiella pneumoniae* 13218 (Table 3). This has not been previously seen in *K. aerogenes* phages. *Klebsiella pneumoniae* is a leading cause of hospital acquired infections and has been linked to a number of illnesses such as wound infections, soft tissue infections, urinary tract infections, pyogenic liver abscess, pneumonia, meningitis, and neonatal sepsis (68). The emergence of multi drug-resistant *Klebsiella pneumoniae* has made it of critical concern for human health and the fight against antibiotic resistance. Phages have been investigated for the treatment of experimentally induced *Klebsiella pneumoniae* burn wound infections and pneumonia (69, 70). Phages have also been used to successfully treat a multi-drug resistant *Klebsiella pneumoniae* wound infection (71). Phage N1M2 showed no homology to known jumbo phages *Klebsiella* phage K64-1 and vB_KleM-RaK2 which both infect *Klebsiella* strains.

**Table 3:** Host range of phage N1M2.

**Strain**	**Efficiency of plaquing**
*Klebsiella aerogenes* N1	1 ± 0
*Klebsiella aerogenes* NCIMB 10102	0.667 ± 0.17
*Klebsiella pneumoniae* NCIMB 13218	0.017 ± 0.009
*Enterobacter gergoviae* DPC 6436	-
*Enterobacter cloacae* DPC 6437	-
*Pseudomonas aeruginosa* PAO1	-
*Escherichia coli* 0157 ΔStx	-
*Escherichia coli* APC 106	-
*Acinetobacter johnsonii*	-
*Staphylococcus aureus* DPC 5247	-

*Efficiency of plaquing is represented as a fraction with SEM of 3 separate experiments. If no N1M2 plaques formed on a strain efficiency of plaquing is represented by (-)*.

It is possible that the increased host range of phage N1M2 is related to the presence of tRNA along with the increased number of genes for genome replication, nucleotide metabolism, and host cell-wall lysis (61). For example the entire genome of *Pseudomonas aeruginosa* Phage KZ can be transcribed using its own RNA polymerase with no significant contribution from the host RNA polymerase (72). tRNAs in phages correspond to codons that are more commonly used in the phage than their bacterial host, especially in genes encoding structural proteins. This reduces the phages reliance on the host cell machinery for replication and increases host range (73). The presence of tRNAs for codons that are preferentially expressed in phage N1M2 compared to N1 was apparent in two out of three cases in phage N1M2 (Table 1). Phage N1M2 has three tRNAs for the codons CAT, TCT, and GTT. The codon usage of CAT (for Histidine) by phage N1M2 is less than that of N1, 1.04 and 1.23%, respectively, with a ratio of phage/host of 0.85. Phage N1M2 uses codon TCT (for Serine) at a frequency of 2.13% while N1 uses TCT at a frequency of 0.73% with a ratio of phage/host of 2.92. Phage N1M2 uses codon GTT (for Valine) at a frequency of 3.40% while N1 uses GTT at a frequency of 1.43% with a phage/host ratio of 2.38.

The jumbo phages also have a number of genes involved in nucleotide metabolism such as thymidylate synthase, thymidylate kinase, ribonucleoside diphosphate reductase, NrdB, and dihydrofolate reductase and multiple genes encoding β and divided β′ subunits specific for multisubunit bacterial RNA polymerases and virion associated RNA polymerases (26). These genes can aid or replace the function of host genes. Of these phage N1M2 contained genes for thymidylate synthase (ORF 184), thymidylate kinase (ORF 249 but of low homology), ribonucleotide diphosphate reductase beta subunit NrdB (166), and dihydrofolate reductase (ORF189). Phage N1M2 contained three genes for RNA polymerase β subunits (ORF37, ORF51, and ORF52) and three putative virion-associated RNA polymerase beta subunits (ORF64, ORF222, and ORF223). Phage N1M2 also contained genes for thymidine kinase (ORF196). A putative electron carrier glutaredoxin (ORF253) was present which could support NrdB function. Again all of these genes showed similarity to OBP.

Phage N1M2 applied for 48 h had no significant effect on a 48 h preformed biofilm (Figure 6A). Phage N1M2 could also not stop a biofilm from forming over 48 h (Figure 6E). However, phage N1M2 applied for 72 h reduced a biofilm formed over 48 h and phage N1M2 applied for 48 h reduced a biofilm formed over 24 h (Figures 6C,D). The importance of allowing an adequate time for phage to have an effect can be seen. Hosseinidoust et al. found that after the addition of phage biofilms increased in some cases up to five times that of untreated controls after 24 h but phage treated biofilms at 48 and 72 h were similar to untreated controls (74). They also found that phage did not significantly reduce biofilms until 48 or 72 h after phage application, before this point the phage treated biofilm was equal to or greater than an untreated biofilm. In some circumstances phage treatment can cause an increase in viable cell counts of biofilms. González et al. found that phage treatment, using a phage that did not infect *E. faecium* or *L. plantarum*, of mixed biofilms formed over 5 h significantly increased viable cell counts of *E. faecium* and *L. plantarum* while in 24 h biofilms *L. plantarum* was slightly decreased and *E. faecium* was similar to untreated controls (75). Mixed species biofilms are often more resistant to antibiotics than single species biofilms.

We tested 48 h of phage application to a 48 h mixed species biofilm to determine if the inclusion of another phage could improve the activity of phage N1M2 in a situation where it had previously been ineffective. Phage K alone could effectively reduce *S. aureus* DPC 5237 in a single species biofilm (Figure 7B). The reduction by Phage K of biofilms has been previously seen (76, 77). Phage N1M2 alone could not reduce N1 numbers in a mixed biofilm. This was expected as phage N1M2 alone could not reduce N1 numbers in a single species biofilm. In XTT assays a combination of phage N1M2 and Phage K was more effective than Phage K alone (Figure 7D). Forty eight hour of phage application to a 48 h biofilm was quantified to see if the inclusion of another phage could improve the activity of N1M2 in a situation where it had previously been ineffective (Supplementary Figure 1). Phage K alone could effectively reduce DPC 5247 numbers in a mixed biofilm as expected since it reduced DPC 5237 in a single species biofilm. The reduction by Phage K of biofilms has been previously seen (76, 77). N1M2 alone could not reduce N1 numbers in a mixed biofilm. This was expected as N1M2 alone could not reduce N1 numbers in a single species biofilm under the same conditions. In XTT assays a combination of N1M2 and Phage K was more effective than Phage K alone but this was not seen when quantifying the biofilm. A discrepancy between XTT results and quantification was previously seen in a study of the effect of antibiotics on biofilms (78). For some strains rifampicin significantly reduced cellular viability in a biofilm as measured by plating but not by XTT and vice-versa. In a study of the antimicrobial activity of plant extracts *Bacillus subtilis* growth was reduced by 100% according to XTT assays but only 60% by plating (79). XTT values were from two separate experiments while viable count values were from three separate experiments.

In conclusion, a novel bacteriophage was isolated against a clinically important bacterial pathogen that is of great interest in the fight against antimicrobial resistance. Phage N1M2 was characterized as a jumbo phage with little homology to other *K. aerogenes* phages and jumbo phages. The most related phage was the jumbo phage *Pseudomonas* phage OBP. Interest is growing in the use of phages in place of antibiotics. *K. aerogenes* can be found as a constituent of biofilms on medical devices. Phage N1M2 was found to be effective at reducing preformed biofilms but not in stopping the formation of biofilms. Biofilms on medical devices often contain mixed bacterial communities. *S. aureus* DPC 5247 was used in biofilms in combination with *K. aerogenes* N1. Phage N1M2 in combination with Phage K were significantly better at reducing a pre-formed *K. aerogenes* and *S. aureus* biofilm than either phage alone.

## Data Availability Statement

The datasets generated for this study can be found in NCBI, accession numbers MN642089 and CP047259.

## Ethics Statement

Written informed consent was given according to study protocol APC055, approved by the Cork Research Ethics Committee (CREC).

## Author Contributions

RL, CH, and RR conceived the study. Investigation was carried out by RL. Bioinformatic analysis was carried out by AC, SS, CB, and RL. LD participated in the biofilm assays design and analysis. RL wrote the original manuscript. CH and RR supervised manuscript revisions.

### Conflict of Interest

The authors declare that the research was conducted in the absence of any commercial or financial relationships that could be construed as a potential conflict of interest.

## References

[1] Wommack KE Hill RT Kessel M Russek-Cohen E Colwell RR. Distribution of viruses in the Chesapeake Bay. Appl Environ Microbiol. (1992) 58:2965–70. 10.1128/AEM.58.9.2965-2970.1992 1444409 PMC183034

[2] Shkoporov AN Hill C. Bacteriophages of the human gut: the “known unknown” of the microbiome. Cell Host Microbe. (2019) 25:195–209. 10.1016/j.chom.2019.01.017 30763534

[3] Mills S Shanahan F Stanton C Hill C Coffey A Ross RP. Movers and shakers: influence of bacteriophages in shaping the mammalian gut microbiota. Gut Microbes. (2013) 4:4–16. 10.4161/gmic.22371 23022738 PMC3555884

[4] Norman JM Handley SA Baldridge MT. Disease–specific alterations in the enteric virome in inflammatory bowel disease. Cell. (2015) 160:447–60. 10.1016/j.cell.2015.01.002 25619688 PMC4312520

[5] Guang-Han O Leang-Chung C Vellasamy KM Mariappan V Li-Yen C Vadivelu J. Experimental phage therapy for *Burkholderia pseudomallei* infection. PLoS ONE. (2016) 11:e0158213. 10.1371/journal.pone.0158213 27387381 PMC4936672

[6] Bai J Kim Y-T Ryu S Lee J-H. Biocontrol and rapid detection of food-borne pathogens using bacteriophages and endolysins. Front Microbiol. (2016) 7:474. 10.3389/fmicb.2016.00474 27092128 PMC4824769

[7] Turnbough CL Jr. Discovery of phage display peptide ligands for species-specific detection of *Bacillus* spores. J Microbiol Methods. (2003) 53:263–71. 10.1016/S0167-7012(03)00030-7 12654497

[8] Pogue JM Kaye KS Cohen DA Marchaim D. Appropriate antimicrobial therapy in the era of multidrug-resistant human pathogens. Clin Microbiol Infect. (2015) 21:302–12. 10.1016/j.cmi.2014.12.025 25743999

[9] World Health Organization WHO Global Priority List of Antibiotic-Resistant Bacteria to Guide Research, Discovery, and Development of New Antibiotics Geneva: WHO (2017).

[10] Munson E Carroll KC. An update on the novel genera and species and revised taxonomic status of bacterial organisms described in 2016 and 2017. J Clin Microbiol. (2019) 57:e01181-18. 10.1128/JCM.01181-18 30257907 PMC6355528

[11] Hede K. Antibiotic resistance: an infectious arms race. Nature. (2014) 509:S2. 10.1038/509S2a 24784426

[12] Kutateladze M Adamia R. Phage therapy experience at the Eliava Institute. Médecine Maladies Infectieuses. (2008) 38:426–30. 10.1016/j.medmal.2008.06.023 18687542

[13] Wright A Hawkins CH Änggård EE Harper DR. A controlled clinical trial of a therapeutic bacteriophage preparation in chronic otitis due to antibiotic-resistant *Pseudomonas aeruginosa*; a preliminary report of efficacy. Clin Otolaryngol. (2009) 34:349–57. 10.1111/j.1749-4486.2009.01973.x 19673983

[14] Jault P Leclerc T Jennes S Pirnay JP Que Y-A Resch G. Efficacy and tolerability of a cocktail of bacteriophages to treat burn wounds infected by *Pseudomonas aeruginosa* (PhagoBurn): a randomised, controlled, double-blind phase 1/2 trial. Lancet Infect Dis. (2018) 19:35–45. 10.1016/S1473-3099(18)30482-1 30292481

[15] Sillankorva SM Oliveira H Azeredo J. Bacteriophages and their role in food safety. Int J Microbiol. (2012) 2012:863945. 10.1155/2012/863945 23316235 PMC3536431

[16] Xu J Chen M He L Zhang S Ding T Yao H. Isolation and characterization of a T4-like phage with a relatively wide host range within *Escherichia coli*. J Basic Microbiol. (2015) 56:405–21. 10.1002/jobm.201500440 26697952

[17] Hwang J-Y Kim J-E Song Y-J Park J-H. Safety of using *Escherichia coli* bacteriophages as a sanitizing agent based on inflammatory responses in rats. Food Sci Biotechnol. (2016) 25:355–60. 10.1007/s10068-016-0050-6 30263278 PMC6049376

[18] Jamalludeen N Johnson RP Friendship R Kropinski AM Lingohr EJ Gyles CL. Isolation and characterization of nine bacteriophages that lyse O149 enterotoxigenic *Escherichia coli*. Vet Microbiol. (2007) 124:47–57. 10.1016/j.vetmic.2007.03.028 17560053

[19] Kim SG Jun JW Giri SS Yun S Kim HJ Chi C. Complete genome sequence of *Staphylococcus aureus* bacteriophage pSa-3. Genome Announce. (2017) 5:e00182-17. 10.1128/genomeA.00182-17 28546473 PMC5477386

[20] Dalmasso M De Haas E Neve H Strain R Cousin FJ Stockdale SR. Isolation of a novel phage with activity against *Streptococcus mutans* biofilms. PLOS ONE. (2015) 10:e0138651. 10.1371/journal.pone.0138651 26398909 PMC4580409

[21] Chang H-C Chen C-R Lin J-W Shen G-H Chang K-M Tseng Y-H. Isolation and characterization of novel giant *Stenotrophomonas maltophilia* phage ϕSMA5. Appl Environ Microbiol. (2005) 71:1387–93. 10.1128/AEM.71.3.1387-1393.2005 15746341 PMC1065149

[22] Gong Z Wang M Yang Q Li Z Xia J Gao Y. Isolation and complete genome sequence of a novel *Pseudoalteromonas* phage PH357 from the Yangtze River Estuary. Curr Microbiol. (2017) 74:1–8. 10.1007/s00284-017-1244-8 28424941

[23] Borriss M Helmke E Hanschke R Schweder T. Isolation and characterization of marine psychrophilic phage-host systems from Arctic sea ice. Extremophiles. (2003) 7:377–84. 10.1007/s00792-003-0334-7 12820036

[24] Sauder AB Quinn MR Brouillette A Caruso S Cresawn S Erill I. Genomic characterization and comparison of seven *Myoviridae* bacteriophage infecting *Bacillus thuringiensis*. Virology. (2016) 489:243–51. 10.1016/j.virol.2015.12.012 26773385

[25] Brussow H Fremont M Bruttin A Sidoti J Constable A Fryder V. Detection and classification of *Streptococcus thermophilus* bacteriophages isolated from industrial milk fermentation. Appl Environ Microbiol. (1994) 60:4537–43. 10.1128/AEM.60.12.4537-4543.1994 7811089 PMC202016

[26] Cornelissen A Hardies SC Shaburova OV Krylov VN Mattheus W Kropinski AM. Complete genome sequence of the giant virus OBP and comparative genome analysis of the diverse PhiKZ-related phages. J Virol. (2012) 86:1844–52. 10.1128/JVI.06330-11 22130535 PMC3264338

[27] Percival SL Suleman L Vuotto C Donelli G. Healthcare-associated infections, medical devices and biofilms: risk, tolerance and control. J Med Microbiol. (2015) 64:323–34. 10.1099/jmm.0.000032 25670813

[28] Kaur S Harjai K Chhibber S. *In vivo* assessment of phage and linezolid based implant coatings for treatment of methicillin resistant *S. aureus* (MRSA) Mediated Orthopaedic Device Related Infections. PLoS ONE. (2016) 11:e0157626. 10.1371/journal.pone.0157626 27333300 PMC4917197

[29] Yilmaz C Colak M Yilmaz BC Ersoz G Kutateladze M Gozlugol M. Bacteriophage therapy in implant-related infections: an experimental study. J Bone Joint Surg Am. (2013) 95:117–25. 10.2106/JBJS.K.01135 23324958

[30] Mulzer J Trampuz A Potapov EV. Treatment of chronic left ventricular assist device infection with local application of bacteriophages. Eur J Cardio Thorac Surg. (2019) ezz295. 10.1093/ejcts/ezz295. [Epub ahead of print]. 31651936

[31] Tkhilaishvili T Winkler T Muller M Perka C Trampuz A. Bacteriophages as adjuvant to antibiotics for the treatment of periprosthetic joint infection caused by multidrug-resistant *Pseudomonas aeruginosa*. Antimicrob Agents Chemother. (2019) 64:e00924-19. 10.1128/AAC.00924-19 31527029 PMC7187616

[32] Hola V Ruzicka F Horka M. Microbial diversity in biofilm infections of the urinary tract with the use of sonication techniques. FEMS Immunol Med Microbiol. (2010) 59:525–8. 10.1111/j.1574-695X.2010.00703.x 20602639

[33] Storti A Pizzolitto AC Pizzolitto EL Detection of mixed microbial biofilms on central venous catheters removed from Intensive care Unit Patients. Brazil J Microbiol. (2005) 36:275–80. 10.1590/S1517-83822005000300013

[34] O'flaherty S Ross RP Meaney W Fitzgerald GF Elbreki MF Coffey A. Potential of the polyvalent anti-*Staphylococcus* bacteriophage K for control of antibiotic-resistant *Staphylococci* from hospitals. Appl Environ Microbiol. (2005) 71:1836–42. 10.1128/AEM.71.4.1836-1842.2005 15812009 PMC1082512

[35] Łubowska N Grygorcewicz B Kosznik-Kwaśnicka K Zauszkiewicz-Pawlak A Wegrzyn A Dołegowska B. Characterization of the three new kayviruses and their lytic activity against multidrug-resistant *Staphylococcus aureus*. Microorganisms. (2019) 7:471. 10.3390/microorganisms7100471 31635437 PMC6843549

[36] Sullivan MJ Petty NK Beatson SA. Easyfig: a genome comparison visualizer. Bioinformatics. (2011) 27:1009–10. 10.1093/bioinformatics/btr039 21278367 PMC3065679

[37] Arndt D Grant JR Marcu A Sajed T Pon A Liang Y. PHASTER: a better, faster version of the PHAST phage search tool. Nucleic Acids Res. (2016) 44:W16–21. 10.1093/nar/gkw387 27141966 PMC4987931

[38] Chen IMA Markowitz VM Chu K Palaniappan K Szeto E Pillay M. IMG/M: integrated genome and metagenome comparative data analysis system. Nucleic Acids Res. (2017) 45:D507–16. 10.1093/nar/gkw929 27738135 PMC5210632

[39] Nurk S Meleshko D Korobeynikov A Pevzner PA. metaSPAdes: a new versatile metagenomic assembler. Genome Res. (2017) 27:824–34. 10.1101/gr.213959.116 28298430 PMC5411777

[40] Altschul SF Gish W Miller W Myers EW Lipman DJ. Basic local alignment search tool. J Mol Biol. (1990) 215:403–10. 10.1016/S0022-2836(05)80360-2 2231712

[41] González-Tortuero E Sutton TDS Velayudhan V Shkoporov AN Draper LA Stockdale SR VIGA: a sensitive, precise and automatic *de novo* VIral Genome Annotator. bioRxiv. (2018) 277509 10.1101/277509

[42] Jones P Binns D Chang H-Y Fraser M Li W Mcanulla C. InterProScan 5: genome-scale protein function classification. Bioinformatics. (2014) 30:1236–40. 10.1093/bioinformatics/btu031 24451626 PMC3998142

[43] Krogh A Larsson B Von Heijne G Sonnhammer EL. Predicting transmembrane protein topology with a hidden Markov model: application to complete genomes. J Mol Biol. (2001) 305:567–80. 10.1006/jmbi.2000.4315 11152613

[44] Juncker AS Willenbrock H Von Heijne G Brunak S Nielsen H Krogh A. Prediction of lipoprotein signal peptides in gram-negative bacteria. Protein Sci. (2003) 12:1652–62. 10.1110/ps.0303703 12876315 PMC2323952

[45] Lowe TM Eddy SR. tRNAscan-SE: a program for improved detection of transfer RNA genes in genomic sequence. Nucleic Acids Res. (1997) 25:955–64. 10.1093/nar/25.5.955 9023104 PMC146525

[46] Laslett D Canback B. ARAGORN, a program to detect tRNA genes and tmRNA genes in nucleotide sequences. Nucleic Acids Res. (2004) 32:11–6. 10.1093/nar/gkh152 14704338 PMC373265

[47] Naville M Ghuillot-Gaudeffroy A Marchais A Gautheret D. ARNold: a web tool for the prediction of Rho-independent transcription terminators. RNA Biol. (2011) 8:11–3. 10.4161/rna.8.1.13346 21282983

[48] Zuker M. Mfold web server for nucleic acid folding and hybridization prediction. Nucleic Acids Res. (2003) 31:3406–15. 10.1093/nar/gkg595 12824337 PMC169194

[49] Wernersson R. FeatureExtract–extraction of sequence annotation made easy. Nucleic Acids Res. (2005) 33:W567–9. 10.1093/nar/gki388 15980537 PMC1160149

[50] Bailey TL Elkan C. Fitting a mixture model by expectation maximization to discover motifs in biopolymers. Proc Int Conf Intell Syst Mol Biol. (1994) 2:28–36. 7584402

[51] Petkau A Stuart-Edwards M Stothard P Van Domselaar G. Interactive microbial genome visualization with GView. Bioinformatics. (2010) 26:3125–6. 10.1093/bioinformatics/btq588 20956244 PMC2995121

[52] Kumar S Stecher G Tamura K. MEGA7: molecular evolutionary genetics analysis version 7.0 for bigger datasets. Mol Biol Evol. (2016) 33:1870–4. 10.1093/molbev/msw054 27004904 PMC8210823

[53] Edgar RC. MUSCLE: multiple sequence alignment with high accuracy and high throughput. Nucleic Acids Res. (2004) 32:1792–7. 10.1093/nar/gkh340 15034147 PMC390337

[54] Jones DT Taylor WR Thornton JM. The rapid generation of mutation data matrices from protein sequences. Comput Appl Biosci. (1992) 8:275–82. 10.1093/bioinformatics/8.3.275 1633570

[55] Felsenstein J. Confidence limits on phylogenies: an approach using the bootstrap. Evolution. (1985) 39:783–91. 10.1111/j.1558-5646.1985.tb00420.x 28561359

[56] Göker M Meier-Kolthoff JP. VICTOR: genome-based phylogeny and classification of prokaryotic viruses. Bioinformatics. (2017) 33:3396–404. 10.1093/bioinformatics/btx440 29036289 PMC5860169

[57] Meier-Kolthoff JP Auch AF Klenk HP Goker M. Genome sequence-based species delimitation with confidence intervals and improved distance functions. BMC Bioinformatics. (2013) 14:60. 10.1186/1471-2105-14-60 23432962 PMC3665452

[58] Tunney MM Ramage G Field TR Moriarty TF Storey DG. Rapid colorimetric assay for antimicrobial susceptibility testing of *Pseudomonas aeruginosa*. Antimicrobial Agents Chemother. (2004) 48:1879–81. 10.1128/AAC.48.5.1879-1881.2004 15105149 PMC400562

[59] Laver T Harrison J O'neill PA Moore K Farbos A Paszkiewicz K. Assessing the performance of the Oxford Nanopore Technologies MinION. Biomol Detect Quantificat. (2015) 3:1–8. 10.1016/j.bdq.2015.02.001 26753127 PMC4691839

[60] Bornet C Chollet R Malléa M Chevalier J Davin-Regli A Pagès J-M. Imipenem and expression of multidrug efflux pump in *Enterobacter aerogenes*. Biochem Biophys Res Commun. (2003) 301:985–90. 10.1016/S0006-291X(03)00074-3 12589810

[61] Yuan Y Gao M. Jumbo bacteriophages: an overview. Front Microbiol. (2017) 8:403. 10.3389/fmicb.2017.00403 28352259 PMC5348500

[62] Hertveldt K Lavigne R Pleteneva E Sernova N Kurochkina L Korchevskii R. Genome comparison of *Pseudomonas aeruginosa* large phages. J Mol Biol. (2005) 354:536–45. 10.1016/j.jmb.2005.08.075 16256135

[63] Mesyanzhinov VV Robben J Grymonprez B Kostyuchenko VA Bourkaltseva MV Sykilinda NN The genome of bacteriophage ϕKZ of *Pseudomonas aeruginosa*. J Mol Biol. (2002) 317:1–19. 10.1006/jmbi.2001.5396 11916376

[64] Mishra CK Choi TJ Kang SC. Isolation and characterization of a bacteriophage F20 virulent to *Enterobacter aerogenes*. J Gen Virol. (2012) 93:2310–4. 10.1099/vir.0.043562-0 22764320

[65] Li E Wei X Ma Y Yin Z Li H Lin W. Isolation and characterization of a bacteriophage phiEap-2 infecting multidrug resistant *Enterobacter aerogenes*. Sci Rep. (2016) 6:28338. 10.1038/srep28338 27320081 PMC4913238

[66] Zhao J Zhang Z Tian C Chen X Hu L Wei X. Characterizing the biology of lytic bacteriophage vB_EaeM_ϕEap-3 infecting multidrug-resistant *Enterobacter aerogenes*. Front Microbiol. (2019) 10:420. 10.3389/fmicb.2019.00420 30891025 PMC6412083

[67] Verthe K Possemiers S Boon N Vaneechoutte M Verstraete W. Stability and activity of an *Enterobacter aerogenes*-specific bacteriophage under simulated gastro-intestinal conditions. Appl Microbiol Biotechnol. (2004) 65:465–72. 10.1007/s00253-004-1585-7 14991251

[68] Holt KE Wertheim H Zadoks RN Baker S Whitehouse CA Dance D. Genomic analysis of diversity, population structure, virulence, and antimicrobial resistance in *Klebsiella pneumoniae*, an urgent threat to public health. Proc Natl Acad Sci USA. (2015) 112:E3574–81. 10.1073/pnas.1501049112 26100894 PMC4500264

[69] Chhibber S Kaur S Kumari S. Therapeutic potential of bacteriophage in treating *Klebsiella pneumoniae* B5055-mediated lobar pneumonia in mice. J Med Microbiol. (2008) 57:1508–13. 10.1099/jmm.0.2008/002873-0 19018021

[70] Chadha P Katare OP Chhibber S. *In vivo* efficacy of single phage versus phage cocktail in resolving burn wound infection in BALB/c mice. Microbial Pathog. (2016) 99:68–77. 10.1016/j.micpath.2016.08.001 27498362

[71] Nir-Paz R Gelman D Khouri A Sisson BM Fackler J Alkalay-Oren S. Successful treatment of antibiotic resistant poly-microbial bone infection with bacteriophages and antibiotics combination. Clin Infect Dis. (2019) 69:2015–8. 10.1093/cid/ciz222 30869755

[72] Ceyssens P-J Minakhin L Van Den Bossche A Yakunina M Klimuk E Blasdel B Development of giant bacteriophage φKZ is independent of the host transcription apparatus. J Virol. (2014) 88:10501–10. 10.1128/JVI.01347-14 24965474 PMC4178840

[73] Yoshikawa G Askora A Blanc-Mathieu R Kawasaki T Li Y Nakano M. *Xanthomonas citri* jumbo phage XacN1 exhibits a wide host range and high complement of tRNA genes. Sci Rep. (2018) 8:4486. 10.1038/s41598-018-22239-3 29540765 PMC5852040

[74] Hosseinidoust Z Tufenkji N Van De Ven TGM. Formation of biofilms under phage predation: considerations concerning a biofilm increase. Biofouling. (2013) 29:457–68. 10.1080/08927014.2013.779370 23597188

[75] González S Fernández L Campelo AB Gutiérrez D Martínez B Rodríguez A. The behavior of *Staphylococcus aureus* dual-species biofilms treated with bacteriophage phiIPLA-RODI depends on the accompanying microorganism. Appl Environ Microbiol. (2017) 83:e02821–16. 10.1128/AEM.02821-16 27836851 PMC5244312

[76] Cerca N Oliveira R Azeredo J. Susceptibility of *Staphylococcus epidermidis* planktonic cells and biofilms to the lytic action of staphylococcus bacteriophage K. Lett Appl Microbiol. (2007) 45:313–7. 10.1111/j.1472-765X.2007.02190.x 17718845

[77] Lungren MP Christensen D Kankotia R Falk I Paxton BE Kim CY *Bacteriophage* K for reduction of *Staphylococcus aureus* biofilm on central venous catheter material. Bacteriophage. (2013) 3:e26825 10.4161/bact.26825 24265979 PMC3829956

[78] Cerca N Martins S Cerca F Jefferson KK Pier GB Oliveira R Comparative assessment of antibiotic susceptibility of coagulase-negative staphylococci in biofilm versus planktonic culture as assessed by bacterial enumeration or rapid XTT colorimetry. J Antimicrob Chemother. (2005) 56:331–6. 10.1093/jac/dki217 15980094 PMC1317301

[79] Al-Bakri AG Afifi FU. Evaluation of antimicrobial activity of selected plant extracts by rapid XTT colorimetry and bacterial enumeration. J Microbiol Methods. (2007) 68:19–25. 10.1016/j.mimet.2006.05.013 16831479

