# Supplementary material for: Isolation of a Novel Jumbo Bacteriophage Effective Against Klebsiella aerogenes
Source: Front Med (Lausanne). 2020 Feb 28;7:67. doi: 10.3389/fmed.2020.00067 (PMC7058600; doi:10.3389/fmed.2020.00067)
Supplement: Supplementary file 1 [file Data_Sheet_1.docx]

Supplementary Material

# Supplementary Figures and Tables

Supplementary table 1. A bank of known strains was selected to be used for phage screening.

| Strain designation | Other designations | Strain |
| --- | --- | --- |
| APC 53 | DPC 6085, NCIMB700577, NCDO0577 | *Bacillus cereus* |
| APC 54 | DPC 6086, NCIMB700578, NCDO0578 | *Bacillus cereus* |
| APC 55 | DPC 6087, NCIMB700579, NCDO0579 | *Bacillus cereus* |
| APC 56 | DPC 6088, NCIMB700827, NCDO0827 | *Bacillus cereus* |
| APC 57 | DPC 6089, NCIMB8079, ATCC7004 | *Bacillus cereus* |
| APC 58 | DPC 6334, Bel 17Bc | *Bacillus cereus* |
| APC 59 | DPC 6336, Bel Bc33 | *Bacillus cereus* |
| DPC 8079 |  | *Bacillus cereus* |
| APC 1762 | UCC 5002 | *Bacillus subtilis* |
| APC 1640 |  | *Enterococcus casseliflavus* |
| APC 1039 | EC618 | *Enterococcus faecalis* |
| APC 1749 | DPC5152 | *Enterococcus faecalis* |
| APC 1025 | EC251 | *Enterococcus faecium* |
| APC 1026 | EC289 | *Enterococcus faecium* |
| APC 1029 | EC300 | *Enterococcus faecium* |
| APC 1030 | EC357 | *Enterococcus faecium* |
| APC 1031 | EC520 | *Enterococcus faecium* |
| APC 1032 | EC533 | *Enterococcus faecium* |
| APC 1035 | EC548 | *Enterococcus faecium* |
| APC 1036 | EC562 | *Enterococcus faecium* |
| APC 1038 | EC587 | *Enterococcus faecium* |
| APC 1043 | *EC725* | *Enterococcus faecium* |
| APC 1044 | *EC748* | *Enterococcus faecium* |
| APC 1641 |  | *Enterococcus faecium* |
| *Lactobacillus salivarius* 6482 | | *Lactobacillus salivarius* |
| APC 317 | DPC5245 | *Staphylococcus aureus* |
| APC 993 | ST290 | *Staphylococcus aureus* |
| APC 994 | ST291 | *Staphylococcus aureus* |
| APC 995 | ST295 | *Staphylococcus aureus* |
| APC 996 | ST299 | *Staphylococcus aureus* |
| APC 998 | ST355 | *Staphylococcus aureus* |
| APC 1003 | ST528 | *Staphylococcus aureus* |
| APC 1004 | ST530 | *Staphylococcus aureus* |
| APC 1007 | ST535 | *Staphylococcus aureus* |
| APC 1009 | ST544 | *Staphylococcus aureus* |
| APC 1010 | ST550 | *Staphylococcus aureus* |
| APC 1012 | 35197 | *Staphylococcus aureus* |
| APC 1019 | 25949 | *Staphylococcus aureus* |
| APC 1022 | Newman | *Staphylococcus aureus* |
| APC 1024 | RF122 | *Staphylococcus aureus* |
| APC 1055 | LMG14694, ATCC 13813, CCRC 10787, CCUG 4208, CIP 103227, DSM 2134, JCM 5671, NCFB 1348, NCTC 8181 | *Streptococcus agalactiae* |
| APC 1759 |  | *Streptococcus agalactiae* |
| APC 1755 |  | *Streptococcus dysgalactiae* |
| APC 119 | DPC 6143, 4001 | *Streptococcus mutans* |
| APC 120 | DC 6144, 4021 | *Streptococcus mutans* |
| APC 121 | DPC 6145, 4070 | *Streptococcus mutans* |
| APC 122 | DPC 6150, 4009 | *Streptococcus mutans* |
| APC 123 | DPC 6151, 4030 | *Streptococcus mutans* |
| APC 124 | DPC 6152, 4037 | *Streptococcus mutans* |
| APC 125 | DPC 6153, 4039 | *Streptococcus mutans* |
| APC 126 | DPC 6154, 4040 | *Streptococcus mutans* |
| APC 127 | DPC 6155, 4055 | *Streptococcus mutans* |
| APC 128 | DPC 6156, 4058 | *Streptococcus mutans* |
| APC 129 | DPC 6157, 3007 | *Streptococcus mutans* |
| APC 130 | DPC 6158, 3013 | *Streptococcus mutans* |
| APC 131 | DPC 6159, 3017 | *Streptococcus mutans* |
| APC 132 | DPC 6160, 1038 | *Streptococcus mutans* |
| APC 133 | DPC 6161, 1054 | *Streptococcus mutans* |
| APC 134 | DPC 6162, NCTC10449, ATCC 25175, SIMS, DSM 20523, ATCC 25175, IFO 13955, NCDO 2062 | *Streptococcus mutans* |
| APC 135 | DPC 6543 | *Streptococcus mutans* |
| APC 1756 | DSM 2071 | *Streptococcus pyogenes* |
| APC 1757 | DSM11728 | *Streptococcus pyogenes* |
| APC 1758 | NCDO2381 | *Streptococcus pyogenes* |
| APC 104 | DPC 6009 | *Escherichia coli* |
| APC 105 | DPC 6050 | *Escherichia coli* |
| APC 106 | DPC 6051 | *Escherichia coli* |
| APC 109 | DPC 6054, P1432 | *Escherichia coli* |
| APC 110 | DPC 6055, AR12900 | *Escherichia coli* |
| APC 115 | DPC 6472 | *Escherichia coli* |
| APC 1220 | HM605 | *Escherichia coli* |
| *Escherichia coli* 042 | | *Escherichia coli* |
| *Escherichia coli* Nissle | | *Escherichia coli* |
| *Esherichia coli* UTI89 | | *Escherichia coli* |
| APC 1977 | MG1655 | *Escherichia coli* |
| APC 176 | DPC 6452 | *Salmonella typhimurium* |
| APC 181 | DPC 6547 | *Salmonella typhimurium* |
| APC 185 | DPC 6436 | *Salmonella typhimurium* |

Supplementary table 2. Resistance genes present in *K. aerogenes* N1 draft genome.

| Start | End | Strand | Size (bp) | Protein |
| --- | --- | --- | --- | --- |
| 366872 | 367006 | + | 135 | Multidrug resistance protein MdtM |
| 367003 | 367434 | + | 432 | Multidrug resistance protein MdtM |
| 367400 | 367756 | + | 357 | Multidrug resistance protein MdtM |
| 367741 | 367899 | + | 159 | Multidrug resistance protein MdtM |
| 375134 | 375493 | - | 360 | Fosfomycin resistance protein FosA |
| 395992 | 396282 | + | 291 | Small multidrug resistance family (SMR) |
| 396382 | 396648 | + | 267 | Small multidrug resistance family (SMR) |
| 594242 | 594364 | + | 123 | RND efflux system, membrane fusion protein |
| 594373 | 594609 | + | 237 | RND efflux system, membrane fusion protein |
| 594740 | 594925 | + | 186 | RND efflux system, membrane fusion protein |
| 595140 | 595373 | + | 234 | Bacteriocin/lantibiotic efflux ABC transporter, permease/ATP-binding protein |
| 595333 | 595560 | + | 228 | Bacteriocin/lantibiotic efflux ABC transporter, permease/ATP-binding protein |
| 595535 | 595828 | + | 294 | Bacteriocin/lantibiotic efflux ABC transporter, permease/ATP-binding protein |
| 595825 | 596058 | + | 234 | Bacteriocin/lantibiotic efflux ABC transporter, permease/ATP-binding protein |
| 596018 | 596341 | + | 324 | Bacteriocin/lantibiotic efflux ABC transporter, permease/ATP-binding protein |
| 596494 | 597003 | + | 510 | Bacteriocin/lantibiotic efflux ABC transporter, permease/ATP-binding protein |
| 596996 | 597163 | + | 168 | Bacteriocin/lantibiotic efflux ABC transporter, permease/ATP-binding protein |
| 1013381 | 1013539 | - | 159 | Multidrug resistance protein MdtL |
| 1013536 | 1013667 | - | 132 | Multidrug resistance protein MdtL |
| 1013682 | 1014032 | - | 351 | Multidrug resistance protein MdtL |
| 1014085 | 1014483 | - | 399 | Multidrug resistance protein MdtL |
| 1062821 | 1063504 | - | 684 | Multidrug resistance protein EmrD |
| 1063461 | 1063679 | - | 219 | Multidrug resistance protein EmrD |
| 1063631 | 1063930 | - | 300 | Multidrug resistance protein EmrD |
| 1092936 | 1093298 | - | 363 | Drug resistance transporter EmrB/QacA subfamily |
| 1093298 | 1093591 | - | 294 | Drug resistance transporter EmrB/QacA subfamily |
| 1096061 | 1096552 | - | 492 | RND efflux system, membrane fusion protein |
| 1096673 | 1096924 | + | 252 | RND efflux system, membrane fusion protein |
| 1096899 | 1097234 | + | 336 | RND efflux system, membrane fusion protein |
| 1097188 | 1097952 | + | 765 | RND efflux system, inner membrane transporter |
| 1097989 | 1098135 | + | 147 | RND efflux system, inner membrane transporter |
| 1098149 | 1098739 | + | 591 | RND efflux system, inner membrane transporter |
| 1098711 | 1099190 | + | 480 | RND efflux system, inner membrane transporter |
| 1099212 | 1099739 | + | 528 | RND efflux system, inner membrane transporter |
| 1099718 | 1100335 | + | 618 | RND efflux system, inner membrane transporter |
| 1232604 | 1232894 | + | 291 | Putative resistance protein |
| 1232860 | 1233060 | + | 201 | Putative resistance protein |
| 1233035 | 1233139 | + | 105 | Putative resistance protein |
| 1233139 | 1233561 | + | 423 | Putative resistance protein |
| 1233524 | 1233646 | + | 123 | Putative resistance protein |
| 1233697 | 1233840 | + | 144 | Putative resistance protein |
| 1487046 | 1487204 | - | 159 | RND efflux system, inner membrane transporter CmeB |
| 1487295 | 1488350 | - | 1056 | Multidrug efflux system AcrEF-TolC, inner-membrane proton/drug antiporter AcrF (RND type) |
| 1488379 | 1488789 | - | 411 | Multidrug efflux system AcrEF-TolC, inner-membrane proton/drug antiporter AcrF (RND type) |
| 1488756 | 1489157 | - | 402 | Multidrug efflux system AcrEF-TolC, inner-membrane proton/drug antiporter AcrF (RND type) |
| 1489157 | 1489447 | - | 291 | Multidrug efflux system AcrEF-TolC, inner-membrane proton/drug antiporter AcrF (RND type) |
| 1489545 | 1489910 | - | 366 | Multidrug efflux system AcrEF-TolC, inner-membrane proton/drug antiporter AcrF (RND type) |
| 1490001 | 1490222 | - | 222 | Multidrug efflux system AcrEF-TolC, inner-membrane proton/drug antiporter AcrF (RND type) |
| 1490236 | 1490601 | - | 366 | Multidrug efflux system AcrEF-TolC, membrane fusion component AcrE |
| 1490598 | 1490858 | - | 261 | Multidrug efflux system AcrEF-TolC, membrane fusion component AcrE |
| 1490827 | 1491378 | - | 552 | Multidrug efflux system AcrEF-TolC, membrane fusion component AcrE |
| 1943717 | 1944244 | - | 528 | Multidrug resistance outer membrane protein MdtP |
| 1944216 | 1944545 | - | 330 | Multidrug resistance outer membrane protein MdtP |
| 1944565 | 1944981 | - | 417 | Multidrug resistance outer membrane protein MdtP |
| 1977822 | 1978478 | + | 657 | Permease of the drug/metabolite transporter (DMT) superfamily |
| 2167705 | 2168190 | - | 486 | Multidrug efflux system EmrAB-OMF, inner-membrane proton/drug antiporter EmrB (MFS type) |
| 2168181 | 2168408 | - | 228 | Multidrug efflux system EmrAB-OMF, inner-membrane proton/drug antiporter EmrB (MFS type) |
| 2168384 | 2168506 | - | 123 | Multidrug efflux system EmrAB-OMF, inner-membrane proton/drug antiporter EmrB (MFS type) |
| 2168559 | 2168675 | - | 117 | Multidrug efflux system EmrAB-OMF, inner-membrane proton/drug antiporter EmrB (MFS type) |
| 2168659 | 2169288 | - | 630 | Multidrug efflux system EmrAB-OMF, inner-membrane proton/drug antiporter EmrB (MFS type) |
| 2169588 | 2170187 | - | 600 | Multidrug efflux system EmrAB-OMF, membrane fusion component EmrA |
| 2170301 | 2170513 | - | 213 | Multidrug efflux system EmrAB-OMF, membrane fusion component EmrA |
| 2170656 | 2170772 | - | 117 | Multidrug resistance regulator EmrR |
| 2170931 | 2171053 | - | 123 | Multidrug resistance regulator EmrR |
| 2171077 | 2171268 | - | 192 | Multidrug resistance regulator EmrR |
| 2206785 | 2207132 | + | 348 | Multidrug resistance protein ErmB |
| 2207174 | 2207566 | + | 393 | Multidrug resistance protein ErmB |
| 2207603 | 2207902 | + | 300 | Multidrug resistance protein ErmB |
| 2395428 | 2395754 | - | 327 | Aminoglycosides efflux system AcrAD-TolC, inner-membrane proton/drug antiporter AcrD (RND type) |
| 2395871 | 2396503 | - | 633 | Aminoglycosides efflux system AcrAD-TolC, inner-membrane proton/drug antiporter AcrD (RND type) |
| 2396461 | 2397096 | - | 636 | Aminoglycosides efflux system AcrAD-TolC, inner-membrane proton/drug antiporter AcrD (RND type) |
| 2397099 | 2397434 | - | 336 | Aminoglycosides efflux system AcrAD-TolC, inner-membrane proton/drug antiporter AcrD (RND type) |
| 2397455 | 2397658 | - | 204 | Aminoglycosides efflux system AcrAD-TolC, inner-membrane proton/drug antiporter AcrD (RND type) |
| 2397721 | 2398134 | - | 414 | Aminoglycosides efflux system AcrAD-TolC, inner-membrane proton/drug antiporter AcrD (RND type) |
| 2398107 | 2398478 | - | 372 | Aminoglycosides efflux system AcrAD-TolC, inner-membrane proton/drug antiporter AcrD (RND type) |
| 2630196 | 2630564 | + | 369 | Multidrug resistance transporter - Bicyclomycin resistance protein Bcr |
| 2630564 | 2630755 | + | 192 | Multidrug resistance transporter - Bicyclomycin resistance protein Bcr |
| 2630736 | 2630849 | + | 114 | Multidrug resistance transporter - Bicyclomycin resistance protein Bcr |
| 2630876 | 2631007 | + | 132 | Multidrug resistance transporter - Bicyclomycin resistance protein Bcr |
| 2630979 | 2631503 | + | 525 | Multidrug resistance transporter - Bicyclomycin resistance protein Bcr |
| 2685749 | 2686591 | + | 843 | Putative multidrug resistance outer membrane protein MdtQ |
| 2686554 | 2686691 | + | 138 | Putative multidrug resistance outer membrane protein MdtQ |
| 2686721 | 2686951 | + | 231 | Putative multidrug resistance outer membrane protein MdtQ |
| 2739525 | 2740010 | - | 486 | Multidrug efflux system MdtABC-TolC, inner-membrane proton/drug antiporter MdtC (RND type) |
| 2739991 | 2740539 | - | 549 | Multidrug efflux system MdtABC-TolC, inner-membrane proton/drug antiporter MdtC (RND type) |
| 2740586 | 2740711 | - | 126 | Multidrug efflux system MdtABC-TolC, inner-membrane proton/drug antiporter MdtC (RND type) |
| 2740689 | 2740826 | - | 141 | Multidrug efflux system MdtABC-TolC, inner-membrane proton/drug antiporter MdtC (RND type) |
| 2740831 | 2741127 | - | 297 | Multidrug efflux system MdtABC-TolC, inner-membrane proton/drug antiporter MdtC (RND type) |
| 2741124 | 2741768 | - | 645 | Multidrug efflux system MdtABC-TolC, inner-membrane proton/drug antiporter MdtC (RND type) |
| 2741807 | 2741935 | - | 129 | Multidrug efflux system MdtABC-TolC, inner-membrane proton/drug antiporter MdtC (RND type) |
| 2741919 | 2742203 | - | 285 | Multidrug efflux system MdtABC-TolC, inner-membrane proton/drug antiporter MdtC (RND type) |
| 2742223 | 2742621 | - | 399 | Multidrug efflux system MdtABC-TolC, inner-membrane proton/drug antiporter MdtC (RND type) |
| 2742714 | 2742935 | - | 222 | Multidrug efflux system MdtABC-TolC, inner-membrane proton/drug antiporter MdtC (RND type) |
| 2742932 | 2743837 | - | 906 | Multidrug efflux system MdtABC-TolC, inner-membrane proton/drug antiporter MdtC (RND type) |
| 2743851 | 2744522 | - | 672 | Multidrug efflux system MdtABC-TolC, inner-membrane proton/drug antiporter MdtC (RND type) |
| 2744562 | 2745701 | - | 1140 | Multidrug efflux system MdtABC-TolC, inner-membrane proton/drug antiporter MdtC (RND type) |
| 2745674 | 2746384 | - | 711 | Multidrug efflux system MdtABC-TolC, membrane fusion component MdtA |
| 2746392 | 2746817 | - | 426 | Multidrug efflux system MdtABC-TolC, membrane fusion component MdtA |
| 3408656 | 3408946 | - | 291 | Permease of the drug/metabolite transporter (DMT) superfamily |
| 3408960 | 3409220 | - | 261 | Permease of the drug/metabolite transporter (DMT) superfamily |
| 3409183 | 3409347 | - | 165 | Permease of the drug/metabolite transporter (DMT) superfamily |
| 3621902 | 3622261 | + | 360 | Permease of the drug/metabolite transporter (DMT) superfamily |
| 3622271 | 3622723 | + | 453 | Permease of the drug/metabolite transporter (DMT) superfamily |
| 3653646 | 3653867 | + | 222 | Multiple antibiotic resistance protein MarR |
| 3654038 | 3654274 | + | 237 | Multiple antibiotic resistance protein MarA |
| 3654229 | 3654357 | + | 129 | Multiple antibiotic resistance protein MarA |
| 3654414 | 3654563 | + | 150 | Multiple antibiotic resistance protein MarB |
| 3654560 | 3655003 | - | 444 | Permease of the drug/metabolite transporter (DMT) superfamily |
| 3654981 | 3655451 | - | 471 | Permease of the drug/metabolite transporter (DMT) superfamily |
| 3812304 | 3812795 | + | 492 | Multidrug efflux transporter MdtK/NorM (MATE family) |
| 3812863 | 3813018 | + | 156 | Multidrug efflux transporter MdtK/NorM (MATE family) |
| 3813088 | 3813321 | + | 234 | Multidrug efflux transporter MdtK/NorM (MATE family) |
| 3813334 | 3813690 | + | 357 | Multidrug efflux transporter MdtK/NorM (MATE family) |
| 3910878 | 3911066 | + | 189 | RND efflux system, inner membrane transporter |
| 3996022 | 3996360 | + | 339 | Small multidrug resistance family |
| 4022413 | 4022739 | + | 327 | Permease of the drug/metabolite transporter (DMT) superfamily |
| 4022724 | 4023047 | + | 324 | Permease of the drug/metabolite transporter (DMT) superfamily |
| 4023162 | 4023290 | + | 129 | Permease of the drug/metabolite transporter (DMT) superfamily |
| 4096282 | 4096521 | - | 240 | RND efflux system, inner membrane transporter |
| 4069566 | 4070015 | - | 450 | RND efflux system, inner membrane transporter |
| 4069984 | 4070094 | - | 111 | RND efflux system, inner membrane transporter |
| 4070094 | 4070222 | - | 129 | RND efflux system, inner membrane transporter |
| 4070203 | 4071120 | - | 918 | RND efflux system, inner membrane transporter |
| 4071172 | 4071354 | - | 183 | RND efflux system, inner membrane transporter |
| 4071315 | 4071602 | - | 288 | RND efflux system, inner membrane transporter |
| 4071605 | 4071736 | - | 132 | RND efflux system, inner membrane transporter |
| 4071733 | 4071879 | - | 147 | RND efflux system, inner membrane transporter |
| 4071940 | 4072116 | - | 177 | RND efflux system, inner membrane transporter |
| 4072182 | 4072328 | - | 147 | RND efflux system, inner membrane transporter |
| 4072312 | 4072449 | - | 138 | RND efflux system, inner membrane transporter |
| 4072517 | 4072813 | - | 297 | RND efflux system, membrane fusion protein |
| 4072837 | 4072959 | - | 123 | RND efflux system, membrane fusion protein |
| 4072996 | 4073124 | - | 129 | RND efflux system, membrane fusion protein |
| 4073121 | 4073657 | - | 537 | RND efflux system, membrane fusion protein |
| 4150746 | 4150934 | + | 189 | Multidrug resistance protein MdtH |
| 4150895 | 4151170 | + | 276 | Multidrug resistance protein MdtH |
| 4151131 | 4151865 | + | 735 | Multidrug resistance protein MdtH |
| 4158614 | 4158748 | + | 135 | Multidrug resistance protein MdtG |
| 4158762 | 4159766 | + | 1005 | Multidrug resistance protein MdtG |
| 4195855 | 4196394 | - | 540 | Permease of the drug/metabolite transporter (DMT) superfamily |
| 4195662 | 4196856 | - | 195 | Permease of the drug/metabolite transporter (DMT) superfamily |
| 4471180 | 4471380 | - | 201 | Macrolide-specific efflux protein MacA |
| 4471377 | 4472069 | - | 693 | Macrolide-specific efflux protein MacA |
| 4472123 | 4472251 | - | 129 | Macrolide-specific efflux protein MacA |
| 4519566 | 4519937 | - | 372 | Multidrug translocase MdfA |
| 4519948 | 4520121 | - | 174 | Multidrug translocase MdfA |
| 4520106 | 4520213 | - | 108 | Multidrug translocase MdfA |
| 4520210 | 4520509 | - | 300 | Multidrug translocase MdfA |
| 4520542 | 4520946 | - | 405 | Multidrug translocase MdfA |
| 4912248 | 4912634 | + | 387 | Fosmidomycin resistance protein |
| 4912741 | 4913205 | + | 465 | Fosmidomycin resistance protein |
| 4929836 | 4930288 | + | 453 | Multidrug efflux system AcrAB-TolC, membrane fusion component AcrA |
| 4930320 | 4930838 | + | 519 | Multidrug efflux system AcrAB-TolC, membrane fusion component AcrA |
| 4930838 | 4930999 | + | 162 | Multidrug efflux system AcrAB-TolC, membrane fusion component AcrA |
| 4931198 | 4931398 | + | 201 | Multidrug efflux system AcrAB-TolC, inner-membrane proton/drug antiporter AcrB (RND type) |
| 4931395 | 4931748 | + | 354 | Multidrug efflux system AcrAB-TolC, inner-membrane proton/drug antiporter AcrB (RND type) |
| 4931705 | 4932019 | + | 315 | Multidrug efflux system AcrAB-TolC, inner-membrane proton/drug antiporter AcrB (RND type) |
| 4932016 | 4932243 | + | 228 | RND efflux system, inner membrane transporter CmeB |
| 4932251 | 4932913 | + | 663 | Multidrug efflux system AcrAB-TolC, inner-membrane proton/drug antiporter AcrB (RND type) |
| 4932974 | 4933099 | + | 126 | Multidrug efflux system AcrAB-TolC, inner-membrane proton/drug antiporter AcrB (RND type) |
| 4933134 | 4933253 | + | 120 | Multidrug efflux system AcrAB-TolC, inner-membrane proton/drug antiporter AcrB (RND type) |
| 4933324 | 4933905 | + | 582 | Multidrug efflux system AcrAB-TolC, inner-membrane proton/drug antiporter AcrB (RND type) |
| 4933932 | 4934138 | + | 207 | Multidrug efflux system AcrAB-TolC, inner-membrane proton/drug antiporter AcrB (RND type) |

Supplementary table 3. ORFS table N1M2. Genes are colour coded. Structural proteins are shown in yellow, DNA replication genes are shown in blue, DNA recombination and repair genes are shown in green, Nucleotide metabolism genes are shown in peach, and Lysis genes are shown in gold.

Supplementary table 4. Predicted promotors in *Klebsiella aerogenes* phage N1M2 shared with Pseudomonas phage OBP.

| Promoter | Start | End | Sequence | E value |
| --- | --- | --- | --- | --- |
|  |  |  | **TBYAWWWWWTTTCARRYAKATATTATYWAAGTGWA** | 3.5e-093 |
| pORF 9 | 4117 | 4152 | **CTCATCAATAATCAAATCGAGATCTTGAAAATCTT** | 8.9e-5 |
| pORF 14 | 6381 | 6417 | **CTTATTTGTTTTGAAATAGATATTATTAGATTGAA** | 1.6e-10 |
| pORF 24 | 12701 | 12735 | **GTTATAAAATTTCAAACAAATATTATCCTCATGTA** | 7.4e-12 |
| pORF 41 | 26928 | 26963 | **TGTATTTCATTTCAGACCTATATTACTTAAGTGAT** | 1.0e-12 |
| pORF 47 | 31335 | 31370 | **TCTAAAAATGTTTAGGCAGATATTATTAGAGTGTA** | 3.1e-14 |
| pORF 50 | 36515 complement | 36550 | **TACATATAGATAATATCTGTCTGATTATCTTTGGC** | 5.5e-13 |
| pORF 51 | 36515 | 36550 | **GCCAAAGATAATCAGACAGATATTATCTATATGTA** | 5.5e-13 |
| pORF 56 | 41620 | 41655 | **GTAATAAATTTTTAAGTAGATATTATTTATAGGAG** | 3.0e-10 |
| pORF 70 | 53113 | 53149 | **TCTATTGATTTTCAAGCAGATATTATTAACATGTA** | 4.6e-15 |
| pORF 85 | 69322 | 69357 | **AACAATTTAATTAAATCGTTTTAACTCTTTCTATA** | 4.0e-6 |
| pORF 104 | 90554 | 90589 | **TGAAAGAAATTTCAGGTCTATATCATCTAAGTGTA** | 6.8e-12 |
| pORF 122 | 120146 | 120181 | **GTCACATATTTTCAGGCAGATATTATCATTATGTA** | 2.8e-13 |
| pORF 131 | 129052 | 129087 | **TGCATATAAGTTTAAGCAGATATTATCACAGTGTA** | 2.7e-14 |
| pORF 149 | 138627 | 138662 | **TGTATTTATTTTTGGACTTATATTATCTAAGTGTA** | 1.7e-12 |
| pORF 167 | 166693 | 166728 | **TCCAAATCTTTTCAGGTAGATATTATTAAAGTGAA** | 8.6e-16 |
| pORF 168 | 167710 | 167745 | **TGCATTTCATTTTAAACCGATATTATTAACGTGAG** | 2.3e-13 |
| pORF 179 | 173383 complement | 173418 | **TACATAATGATAATATCTGTCTATTTTTATTTGAA** | 2.1e-11 |
| pORF 180 | 173383 | 173418 | **TTCAAATAAAAATAGACAGATATTATCATTATGTA** | 2.1e-11 |
| pORF 187 | 177684 | 177720 | **TGTATTTCTTTTCAGACATATATAATTAAAGTGTA** | 8.5e-15 |
| pORF 192 | 179906 | 179941 | **GGTAAAATTTTTCAAACAGATATTATCTAAGTAGT** | 4.0e-12 |
| pORF 197 | 183895 | 183930 | **TGTATCTTATTTCAGACATATATCATTTAAGTGAA** | 2.5e-13 |
| pORF 199 | 185535 | 185570 | **TCTATTTTAAAACAGATAGATATTATCATAGTGTA** | 6.1e-13 |
| pORF 216 | 201530 complement | 201565 | **TCTACTTTGATAATATTTGTTTGAAATATAATAGT** | 3.4e-11 |
| pORF 217 | 201530 | 201565 | **ACTATTATATTTCAAACAAATATTATCAAAGTAGA** | 3.4e-11 |
| pORF 219 | 202605 | 202640 | **CTCTTTTAAATAATTTATTTTTTTTTAGGATTAAT** | 3.7e-5 |
| pORF 230 | 224133 | 224168 | **GCTATTTAATTTCAAGCAGATATTATTAACGTGTT** | 2.0e-15 |
| pORF 233 | 225820 | 225854 | **GCCAAAATGCTTCAGGTATATATTATTACCGTGAA** | 1.3e-11 |
| pORF 239 | 228726 | 228761 | **TACATTCATAGTCAGGTCTATATTATCTAAGTGTA** | 1.0e-11 |
| pORF 240 | 229336 | 229371 | **TGAATACAAATTCAGAAATATATAATCTAATTGAT** | 3.2e-10 |
| pORF 242 | 230861 | 230895 | **TTTAAAATTATTCAGACGTATATTATTAGCATGTA** | 4.0e-12 |
| pORF 256 | 252179 | 252214 | **TCCATTATTAAATAAATAGATATTATTAGATTGAA** | 6.0e-11 |

Supplementary table 5. Terminators of Phage N1M2 detected using ARNold and confirmed using Mfold Quikfold. Loops and stems are shown in red and blue respectively.

| Terminator | Start | End | Sequence | ΔG kcal/mol |
| --- | --- | --- | --- | --- |
| tORF5 | 2731 | 2770 | GGGGTGGTGGGTAAAACCGTCACCCCgTTTTGATACCAC | -18.60 |
| tORF13 | 6347 | 6384 | CTATCACCTCCAAAAGGGGTGATAGTTTTTAGTTCTT | -14.10 |
| tORF14 | 7153 | 7189 | TTGGTCTGGTGGTTATCAGATCAATTTACTTTCTGG | -10.10 |
| tORF18 | 9195 | 9229 | TGGGGAGGTCAACCCTCCCTAaTTTTAATAGATC | -13.12 |
| tORF27 | 15041 | 15072 | GCCCTGGCTAGTCCAGGGCTTTTTATATTGC | -13.60 |
| tORF32 | 17679 | 17710 | GCGCTCAATACAAGAGCGCTTTGATTACCGG | -10.50 |
| tORF | 23568 complement | 23612 | CAGAGAGGAGGGCTTTCACCCACCCTCTCTGTTAATTTAGTTAA | -17.20 |
| tORF36 | 23582 | 23625 | CAGAGAGGGTGGGTGAAAGCCCTCCTCTCTGTTGTTTTTATAGG | -23.20 |
| tORF38 | 25903 | 25941 | GCAATGGGGTGGAAACACCCTGTTGCTTTTATCAGGTT | -21.40 |
| tORF | 27171complement | 27213 | CAGGATCTCCCGGTTAGGGGAGATCCTGgTTATGACTTATTT | -22.00 |
| tORF41 | 27185 | 27226 | CAGGATCTCCCCTAACCGGGAGATCCTGTTATTCTATACTT | -20.60 |
| tORF50 | 36095 complement | 36135 | TACCACCTACTATAGAGTAGGTGGTAgTTATTTACATAAA | -17.40 |
| tORF60 | 45037 | 45076 | CTTACCCTTCCTAGTGGAGGGTAAGggTTATATGTTTAT | -14.80 |
| tORF | 46140 complement | 46179 | CTACTCACCCTTAATTGGGTGAGTAGgTATGTGTTATTT | -18.90 |
| tORF64 | 47405 complement | 47446 | CCTCTACTCCCGTGATGGGAGTAGAGGTTATGTTTACTTA | -20.60 |
| tORF63 | 47419 | 47457 | CTCTACTCCCATCACGGGAGTAGAGgTCTGTTATGCTA | -18.40 |
| tORF67 | 52161 | 52193 | GCCGTCTTAGAAAGGCGGCTTTTCATGATCA | -11.10 |
| tORF73 | 54535 complement | 54578 | CCCTACCCACTCCGAAAGGAGGGGTAGGGgTTATGTTTGTTAT | -27.00 |
| tORF85 | 70629 | 70667 | GAGTAGGGGCCTTCGGGCTCCTACTCTTATATGTTTGC | -24.10 |
| tORF87 | 72330 | 72366 | GCTTGGCTGCTAAACAAGCTAAGCTATCTGTTAATG | -12.00 |
| tORF90 | 74598 | 74637 | CTCTACTCCTTTTATCGGGAGTAGGGgTTTGATGTTATG | -15.10 |
| tORF | 94012 | 94044 | CAGGTCATTATTGGCCTGaaTGTTATTACTGA | -10.20 |
| tORF108 | 97725 | 97671 | GACCACGGTTTCGGCCGTGGTCagTTCTATTTATGA | -17.10 |
| tORF109 | 98249 | 98288 | TGTATGGGGGTTATAAAACCCATGCAcTTTTTGTAGGAT | -11.30 |
| tORF111 | 99509 | 99554 | CCCTACCCCTCCAATTAAGGATGGGGTAGGGgTTATGCTTACTAT | -23.50 |
| tORF | 109916 complement | 109945 | GGGGAGTTTCCTCCCCaTTCTATTAACCT | -11.50 |
| tORF117 | 109929 | 109957 | GGGGAGGAAACTCCCCTTTCTTATTTAG | -15.40 |
| tORF119 | 115859 complement | 115903 | CCCCTACCCCTCCGAAAGGAAGGGTAGGGAgTTTATGGTTTATA | -24.50 |
| tORF118 | 115875 | 115915 | CCTACCCTTCCTTTCGGAGGGGTAGGggTTATGTTTACTT | -21.00 |
| tORF120 | 119412 | 119438 | CCCCGGAAACGGGGTTTTATTCATTA | -12.70 |
| tORF125 | 124134 | 127172 | TAGGAGAGGGGAAACTCTCTCCTAcaTTTTTGTTTATA | -19.10 |
| tORF127 | 125095 | 125135 | CCTAGAGAGGGAATTCCCTCTCTAGGacTTTATATTGTTT | -22.00 |
| tORF | 134705 complement | 134741 | GAACCTACCTTAATCGGTAGGTTCTCTATTATGGTT | -14.90 |
| tORF | 14139 complement | 141438 | TGGGGGTTGGTTTTACCCAACCCTTGaTTCTTTCTAATA | -13.40 |
| tORF161 | 150437 | 150467 | GGGGACCGGAGTCCCCCCTTTTACATCAAC | -10.70 |
| tORF163 | 160772 complement | 160813 | TGAGAGTCTGGGGTAACCTGGGCTCTCATTATTATGGTATG | -19.50 |
| tORF164 | 163667 complement | 163705 | AGTCCCTGGGTTCGTCCAGGGACTcTTTATCAGATTCT | -18.90 |
| tORF165 | 164443 complement | 164479 | GTAGGGTGGCCGAAGCCACCCTACTCTATGTTCGGA | -19.80 |
| tORF171 | 169645 | 169686 | AGAGAGGCTTCCGAAAGGAGGTCTCTCTcTTTTTGTACCTGA | -22.70 |
| tORF179 | 172660 complement | 172704 | CCCTACCTAGCCCGCAAAGGCTAGGTAGGGaTTATGCATGTATT | -24.40 |
| tORF184 | 176430 | 176468 | GCTAGGGGCTCTTAGGAGCCTTTAGCTTTTCAAGGTAT | -17.50 |
| tORF | 182639 complement | 182678 | CGAGCTGAGGTGTTACCCTCAGCTCGaTCTTAGTTATGC | -19.00 |
| tORF | 184319 complement | 184355 | CAGGCTACCCGAAGGTAGCCTGagTTTACTTATTCG | -16.30 |
| tORF197 | 184344 | 184638 | AGGCTACCTTCGGGTAGCCTgTTATTCGGTTCTT | -17.10 |
| tORF203 | 187904 complement | 187944 | CCTACCCTTCCTTACGGAGGGGTAGGggTATTTTGTCGTT | -21.00 |
| tORF206 | 191881 | 191920 | CTTACCCTCCGCAAGGAAGGGTAAGggTTATATGTTTGT | -18.00 |
| tORF210 | 196236 complement | 196271 | CTTCCTTGCGGAGGGTAAGGGAGTATGTTTGTTAT | -11.30 |
| tORF220 | 203754 complement | 203798 | CCCTTACCCACTCCGTGAGGAGGGGTAAGGGTTATGCTTGTTTA | -27.20 |
| tORF221 | 206022 complement | 206057 | TGAGGGGACTTCGGTCCCCTTAcTTTTAGAGGTAA | -18.10 |
| tORF | 214038 complement | 214070 | CTGTGACGTTATCGTCGCAGTTTTCTACTGAT | -11.60 |
| tORF223 | 219297 complement | 219328 | AGGCCGTCAATGCGGCCTTTATATAGGGAAA | -11.00 |
| tORF | 222630 complement | 222667 | GTTGGGGATATTAAAACCCCCAGCgTTTAATTCATAC | -11.80 |
| tORF228 | 223407 | 223443 | GTAGGGGTCCTTCGGGATCTCTACTTATTTTGTTAT | -18.20 |
| tORF243 | 232501 complement | 232545 | CCCCTACCCACTCCGAAAGGAGGGGTAGGGGTTTTATGTTGTTT | -29.60 |
| tORF | 247875 complement | 247919 | CCCTACTCCCTCCGAAAGGAAGGGGTAGGGgTTTATGTTTGTTT | -24.60 |
| tORF | 251572 complement | 251608 | GCCAGAGAAATGAATCTCTGGCacTTTTAATGTGAT | -14.40 |

A.

B.

Supplementary Figure 1. Effect of N1M2 and Phage K on mixed biofilms formed by N1 *K. aerogenes* and DPC 5247 *S.aureus*. No glucose 48 hr biofilm, 48 hr phage. N1 and DPC 5247 were quantified in the biofilm by dilution and spread plating on UTI ChromoSelect agar. The experiment was carried out once but counts were carried out in duplicate. A. Count of N1 in mixed biofilm formed by N1 and DPC 5247. B. Count of DPC 5247 in mixed biofilm formed by N1 and DPC 5247.
